# Enhanced Performance Monitoring as a Transdiagnostic Risk Marker of the Anxiety and Obsessive–Compulsive Spectrum: The Role of Disorder Category, Clinical Status, Family Risk, and Anxiety Dimensions

**DOI:** 10.1155/da/9505414

**Published:** 2025-04-13

**Authors:** Kai Härpfer, Hannes Per Carsten, Franziska Magdalena Kausche, Anja Riesel

**Affiliations:** Department of Psychology, University of Hamburg, Hamburg 20146, Germany

**Keywords:** anxiety, anxious apprehension, CRN, EEG, ERN, family risk

## Abstract

In this preregistered study, we investigated the relationship between neural correlates of performance monitoring and disorders of the anxiety and obsessive–compulsive spectrum. Specifically, we aimed at understanding the role of disorder category, clinical status, family risk, and the transdiagnostic symptom dimensions of anxious apprehension and anxious arousal. To this end, we measured event-related potentials (ERPs) of performance monitoring (i.e., error-related negativity, ERN, and correct-response negativity, CRN) in a large sample of 156 participants, including groups of patients with obsessive–compulsive disorder, social anxiety disorder, and specific phobia, as well as a naturalistic control group. Contrary to our initial expectations, we did not observe significant differences in ERPs among the clinical groups, nor in comparison to the naturalistic control group. However, after creating a more strictly defined healthy control group, we found larger ERN amplitudes in the specific phobia compared with the healthy control group. In addition, when comparing participants with and without a lifetime clinical diagnosis of any internalizing disorder, regardless of their main diagnosis, as well as when comparing those with or without a family risk for internalizing psychopathology, we observed larger amplitudes for both ERN and CRN. Subsequently, we combined data from this study and a previously published subclinical study to examine the role of transdiagnostic symptom dimensions (i.e., anxious apprehension and anxious arousal) across a wider severity spectrum. In this joint sample of 246 participants, gender emerged as a moderator of the link between anxious apprehension and enhanced performance monitoring. Specifically, women with increasing anxious apprehension exhibited elevated ERN and CRN amplitudes. In conclusion, our study challenges the notion of a disorder-specific link to performance monitoring. Instead, our findings suggest that enhanced performance monitoring is associated with a higher propensity for anxious apprehension and acts as a broad risk marker for internalizing psychopathology, reflecting vulnerability beyond diagnostic borders within the anxiety- and obsessive–compulsive spectrum.

## 1. Introduction

Human learning is deeply rooted in trial-and-error approaches. As such, committing an error is not inherently dangerous; rather, it offers an opportunity to enhance future performance. Through cognitive, motivational, and behavioral adjustments informed by past errors, individuals can continually expand and refine their repertoire of skills and abilities [1–4]. Over the past three decades, neuroscientific research has gained deeper insights into the neural processing of errors by examining the event-related potential (ERP) of the human electroencephalogram (EEG) called error-related negativity (ERN; [5]); or originally referred to as error negativity (*N*_E_; [6]). The ERN is a response-locked fronto-central potential peaking at around 50 ms after erroneous responses. It can be measured over the lifespan in individuals aged 5–80 years [7–10], demonstrating high internal consistency across tasks [11–13]) and good test–retest reliability over intervals of up to 2 years [11, 12, 14, 15]. Another performance associated ERP is the correct-response negativity (CRN; [16, 17]), elicited within a similar time interval after correct responses as the ERN, albeit with a relatively smaller amplitude.

As a result of the long-lasting and ongoing research in this field, an extensive body of theoretically and empirically based frameworks has evolved, aiming at describing the functional role of the ERN within the cognitive system. The ERN was initially conceptualized as a signal of mismatch between the intended and actual response [5, 6], later viewed as an indicator of conflict between two simultaneously activated response tendencies [1, 18] and further expanded to emphasize its role in reinforcement learning [19]. What all three approaches have in common is that they acknowledge the importance of the ERN for cognitive control functions, that the ERN likely signals the need for adjustment in order to fulfill the requirements of a changing environment, and that ERN is the starting point of a potential cascade of processes, ultimately culminating in improved future performance.

Additional informative conclusions for the functional role of the ERN can be derived from its neurobiological underpinnings, with the anterior cingulate cortex (ACC) as one of the main generators of the ERN [20–26]. The ACC consists of a high density of dopaminergic neurons [27, 28] and is widely connected to both “cognitive” prefrontal and “emotion processing” limbic regions [29, 30]. Accordingly, ACC activation is not limited solely to error responses; it extends to instances of fear [31] and pain [32, 33], demonstrating its pivotal regulatory function as an integrating hub of both cognitive and emotional processes [2, 29, 34, 35]. Because of its connections to other brain regions as well as its responsiveness to reward and punishment, the ACC is considered a central player in behavior regulation and the implementation of adaptive adjustments [36].

The involvement of the ACC, and by extension, the ERN, in both cognitive and emotional processes shapes a prevailing perspective on the ERN. The ERN is not only considered as an index of an individuals' capacity (i.e., working memory/executive functions; [37, 38]) and motivation (i.e., reward/punishment; [39, 40]) for task engagement but also as an indicator of an individuals' trait-like sensitivity to internal threats (i.e., poor performance; [36]). This makes the ERN particularly relevant for clinical research, as it holds the potential to enhance our understanding of the etiopathogenesis of mental disorders [41]. Consequently, error monitoring has been included by the Research Domain Criteria Initiative (RDoC), which offers a framework that centers on neurobiological mechanisms involved in mental health and disorders [42–44]. The RDoC matrix lists the ERN as a physiological measure within two domains (i.e., cognitive systems and negative valence systems), reflecting its role as an integrative hub of cognition and emotion.

In fact, altered neural error monitoring has been proposed as a transdiagnostic endophenotype [45] applicable to a range of mental disorders [46–48]. Firstly, previous meta-analytical studies found varying ERN amplitudes along the lines of internalizing and externalizing mental disorders [49, 50], with an enhanced ERN in anxiety disorders [51–53], obsessive–compulsive disorders (OCDs) [54–56], and depression ([57]; but see [50]), while a reduced ERN has been observed in substance use disorder [58, 59], psychosis [60], psychopathy [61], and attention deficit hyperactivity disorder ([54, 62]; but see [63]). Secondly, unaffected participants with a family history of an anxiety, obsessive–compulsive, substance use, or attention deficit hyperactivity disorder showed a similar altered ERN like their (first-degree) relatives [48, 64–67], underscoring that altered error-related brain activity is observable not only in symptomatic individuals but also in those with an increased risk. Thirdly, intervention studies found an unchanged increased ERN, despite the successful treatment of anxiety [68, 69] and obsessive–compulsive symptoms [70, 71], demonstrating its independence of symptomatic state and further strengthening that it rather reflects an underlying feature. Lastly, the trait-like magnitude of the ERN has been shown to be heritable [72, 73]. In summary, there is compelling empirical evidence supporting the notion that enhanced error monitoring, as measured by heightened ERN amplitudes, represents a promising transdiagnostic endophenotype that indicates vulnerability for anxiety and OCDs.

However, evidence from primary studies on elevated error monitoring within the anxiety and obsessive–compulsive spectrum mostly rely on case–control designs comparing a clinical and healthy control group. To the best of our knowledge, only a few studies compared two or more disorder categories with each other and/or a control group (e.g., [68, 74–77]). Anxiety and obsessive-compulsive disorders are highly comorbid and overlap in symptoms, which is why we need studies with multiple clinical groups and the assessment of transdiagnostic symptoms to achieve a more holistic understanding of psychopathology and its neural signatures. Furthermore, healthy participants do not necessarily represent a naturalistic control group with characteristics of the general population, bearing the risk of overestimating differences. In summary, the inclusion of various clinical groups and more naturalistic control groups is essential to enhance the research design, to bolster the validity of findings, to facilitate comparisons, and to contribute to a more comprehensive understanding of the neural correlates of performance monitoring in psychopathology.

In addition, studies examining subclinical symptom levels have reported less consistent findings regarding enhanced ERN amplitudes [53, 78–80] and not all disorder categories within the anxiety and obsessive–compulsive spectrum appear to be characterized by enhanced error monitoring [51]. Enhanced ERN amplitudes have been found in generalized anxiety disorder (GAD; [81, 82]), social anxiety disorder (SAD; [68, 75]), health anxiety [76], and OCD ([54–56]) but not in post-traumatic stress disorder (PTSD; [83, 84]) and specific phobia [85, 86]. Concerning panic disorder, the available literature is limited, with only one study, featuring a relatively small sample size, identifying an enhanced ERN in individuals with a primary diagnosis of panic disorder [87]. Finally and to the best of our knowledge, there is no available literature exploring potential alterations of error monitoring in agoraphobia.

Inconsistent findings between disorder categories may stem from variations in latent symptom dimension profiles inherent to anxiety and OCDs [88, 89]. Specifically, the presence of enhanced error monitoring appears to be closely tied to the dimension of anxious apprehension rather than anxious arousal, a notion supported by primary studies ([82, 85, 86, 90–92]; but see [79, 93]) as well as meta-analyses [52, 53]. Additionally, some of this research suggests that the link between anxious apprehension and enhanced error monitoring is more dominant in women [94]. These transdiagnostic findings are relevant insofar as they might be translated into diagnostic categories, whereby disorders characterized more prominently by anxious apprehension exhibit stronger associations with ERN variation. For instance, GAD, SAD, and OCD appear to be more closely linked to ERN enhancements, in contrast to PTSD and specific phobia. Consequently, a dimensional and transdiagnostic conceptualization of the anxiety and obsessive–compulsive spectrum may provide a more comprehensive understanding of the transdiagnostic anxiety dimensions (i.e., anxious apprehension and anxious arousal), that are associated with enhanced error monitoring, while broad disorder categories are likely to overshadow specific associations.

In this preregistered study (https://osf.io/kxv5h), our primary objective was to gain a more thorough understanding of the observed associations between the ERN and disorders of the anxiety and obsessive–compulsive spectrum, by adopting a transdiagnostic and dimensional approach including various anxiety-related disorder categories and dimensions. To this end, patients with disorders that were likely to have different characteristics in anxious apprehension and anxious arousal were recruited. Specifically, performance monitoring associated ERPs (i.e., ERN and CRN) of 156 participants who were diagnosed with OCD, SAD, or specific phobia (PHOB) as well as control participants (CON) were measured while performing a flanker task. In a subsequent step, we integrated the data from this predominantly clinical sample with data from a subclinical study [95], resulting in a sample of 246 participants with a broad range across the symptom severity spectrum. In our first (preregistered) research question, we aimed to explore the disorder-specific links of the diagnostic groups with alterations in the ERN. We expected that ERN amplitudes of the OCD and SAD group would be larger than those of the PHOB and CON groups. Furthermore, we expected no differences between the OCD and SAD groups as well as between the PHOB and CON groups. In our second (nonpreregistered) research question, we were further interested in the role of clinical status of an internalizing disorder as well as family risk for internalizing psychopathology on the ERN. We anticipated that both clinical status and family risk would be associated with larger ERN amplitudes. The third (preregistered) research question aimed at investigating the dimensional relationships between anxiety dimensions and enhanced ERN amplitudes. We predicted anxious apprehension, but not anxious arousal, would be linked to heightened ERN amplitudes, with this association being stronger for clinical participants. The last (nonpreregistered) research question focused on the moderating role of gender in the relationship between anxious apprehension and ERN enhancement. We hypothesized that this link would be more pronounced for women compared with men. To test the specificity of the effects on the ERN, we investigated the CRN in the same manner as the ERN for all research questions. However, these CRN analyses were fully exploratory, given the limited existing evidence regarding the relationship between anxiety and the CRN [51].

## 2. Method

### 2.1. Participants

Using G^*⁣*^*∗*^^Power, version 3.1.9.7 [96, 97], an a priori sample size calculation was conducted. Based on the results of previous meta-analyses [52, 53, 56], we assumed medium-sized effects in our mixed ANOVA. A within–between interaction of response type (correct, incorrect) × group (OCD, SAD, PHOB, CON) of medium size (Cohen's *f*  > 0.28), indicating a group effect on the ERN but not the CRN, can be detected with a sample size of 36 per group, a power of 80%, and an alpha of 0.05 [98]. Specifications of the sample size calculation can be found in the Supporting Information (Figure S1). Therefore, we recruited 160 participants including individuals with OCD, SAD, PHOB, and control participants (*n* = 40 per group) to ensure a sufficient power while expecting a dropout rate of 10% due to the exclusion criteria.

Our final sample consisted of 156 right-handed participants (*n* = 115 identified as female) aged 18–63 years (*M* = 28.51, *SD* = 8.07). The OCD, SAD, PHOB, and CON group consisted of each 39 participants with a corresponding main diagnosis. Regarding the clinical status of an internalizing disorder and the family risk for internalizing psychopathology, there were clinical participants with family risk (*n* = 88) and without family risk (*n* = 34), as well as nonclinical participants with family risk (*n* = 15) and without family risk (*n* = 19) in our sample. The group with a clinical status of an internalizing disorder encompassed all participants from the OCD, SAD, and PHOB groups as well as five participants from the control group diagnosed with depression, sleeping disorder, PTSD, and/or skin picking. The group with a family risk for internalizing psychopathology encompassed participants with a first-degree relative suffering from at least one disorder of the anxiety and obsessive–compulsive spectrum, depression, skin picking, trichotillomania, tics, and/or body dysmorphia.

Participants were recruited via the outpatient unit of the University of Hamburg, student recruiting systems of the university, campus flyers and posters, as well as advertisements placed in local medical and counselling centers, online peer support forums, social media, and part-time jobs platforms (Facebook). Participants were screened for eligibility and had to fulfil the following criteria: age between 18 and 65 years, at least fluent German language skills, normal or corrected-to-normal vision, and the ability to provide written informed consent. Exclusion criteria for all subjects included a history of any neurological disorder, substance-related disorders (current or lifetime), schizophrenia spectrum disorder (current or lifetime), manic episode in the context of a bipolar disorder (current), use of benzodiazepines during the last week, and neuroleptic medication during the last three months. Further exclusion criteria for the control group included a current or lifetime OCD, SAD, or specific phobia. A detailed decomposition of each group's clinical diagnoses is listed in the Supporting Information (Table S1). All participants received either course credit or monetary compensation for their study participation.

Prior to data analysis, two participants from the control group were excluded and replaced because they were mistakenly included in the study even though they fulfilled the criteria of a lifetime diagnosis OCD, SAD, or specific phobia. One control participant's study participation was canceled because of an unwillingness to follow experimental instructions and was therefore excluded and replaced. Another control participant was excluded due to a low accuracy in the flanker task (i.e., <50%), likely indicating that this person did not follow the task instruction. And another three participants, each one of the clinical groups, were excluded due to low EEG quality (i.e., >25% artifacts). At the time of participation, 47 participants were currently medicated with at least one drug including antidepressants (*n* = 15), oral contraceptives (*n* = 9), thyroid hormones (*n* = 9), dermatological drugs (*n* = 2), and other drugs of which none was reported more often than by one participant (*n* = 12). However, neither the overall medicated participants (all *p*s > 0.21) nor those using antidepressants (all *p*s > 0.22) exhibited significant differences in ERN or CRN.

### 2.2. Procedure

Prior to the laboratory assessment, participants were screened for eligibility using the inclusion and exclusion criteria specified earlier. They received verbal and written information on the objectives and methods of the study and gave written informed consent. Psychopathological symptoms, severity, and clinical diagnoses were assessed by respective questionnaires and structured interviews by trained personnel. After preparing the EEG, participants underwent an 8-min resting state before performing the flanker task. The study procedure was approved by the local ethics committee of the University of Hamburg (AZ: 2021_351) and was planned as well as conducted in accordance with the Declaration of Helsinki [99].

### 2.3. Measures

Clinical diagnoses were assessed using the Structured Clinical Interview for DSM-5 Disorders – Clinical Version (SCID-5-CV; [100, 101]). Family risk for internalizing psychopathology was derived via the Family History Screen [102]. Obsessive–compulsive symptom severity of OCD patients was determined using the Yale-Brown Obsessive Compulsive Scale (Y-BOCS; [103, 104]). Following these interviews, overall severity of illness was rated based on the Clinical Global Impression Scale (CGI-S; [105]), and global functioning was scored on the global assessment of functioning (GAF; [106]).

In addition, a battery of questionnaires were administered. Obsessive–compulsive symptoms were measured by the Obsessive-Compulsive Inventory Revised (OCI-R; 20 items, 5-point Likert scale 0–4; *α* = 0.91; [107, 108]), social phobia symptoms by the self-report version of the Liebowitz Social Anxiety Scale (LSAS-SR; 24 items, 4-point Likert scale 0–3; *α* = 0.98; [109, 110]), specific phobia symptoms by the Severity Measure for Specific Phobia (SMSP; 10 items, 5-point Likert scale 0–4; *α* = 0.85; [111]), anxious apprehension by the Penn State Worry Questionnaire (PSWQ; 16 items, 5-point Likert scale 1–5; *α* = 0.94; [112, 113]), anxious arousal by the respective subscale of the Mood and Anxiety Symptom Questionnaire (MASQ-AA; 17 items, 5-point Likert scale 1–5; *α* = 0.89; [114, 115]), state and trait anxiety by the respective subscales of the State–Trait-Anxiety Inventory (STAI-S and STAI-T; each 20 items, 4-point Likert scale 1–4; *α* = 0.95 and *α* = 0.95; [116, 117]), depression symptoms by the Beck Depression Inventory (BDI-II; 21 items, 4-point Likert scale 0–3; *α* = 0.95; [118, 119]), alcohol consumption by the Alcohol Use Disorders Identification Test (AUDIT; 10 items, 5-point Likert scale 0–4; *α* = 0.70; [120]), and handedness by the modified Edinburgh Handedness Inventory (EHI; 10 items, 5-point Likert scale −10 to + 10; *α* = 0.92; [121, 122]). Additional questionnaires were administered for the purpose of investigating other research questions (https://osf.io/zrm2j), and results will be reported elsewhere.

### 2.4. Flanker Task

Participants performed a speeded arrowhead version of the flanker task [123] while they were sitting in a dimly lit, electrically shielded cabin ~24 in. in front of a 19-inch LCD monitor with a resolution 1920 × 1080 pixels and a refresh rate of 120 Hz. The task was identical with the one we previously used in another study [95] and consisted of a set of five horizontally aligned arrows (one target, four flankers) displayed by Presentation Software (Neurobehavioral Systems, Inc., Albany, California). The set of arrows was ~6.2° in width and ~1.0° in height with an equal number of trials pointing pseudo randomly either into same (<<<<< or >>>>>) or opposite directions (<<><< or >><>>). Participants were instructed to indicate the direction of the center arrow by pressing a key with their respective left or right index finger as quickly and accurately as possible, which was practiced in 20 trials at the beginning of the task. Each trial started with a fixation (200–1200 ms), followed by the presentation of arrow stimuli (100 ms) and a response window (max. 800 ms). The task consisted of five blocks with each 80 trials equaling 400 trials in total and participants received a performance feedback after each block asking them to respond faster, irrespective of their actual response times. The speed performance feedback was implemented to promote a sufficient amount of error trials, to reduce the length of the task to a reasonable extent, and to increase discrimination between groups since group differences of the ERN between OCD patients and healthy control participants were found to be pronounced under speed conditions [39].

Accuracy was defined as the percentage of correct responses of the response trials, response times as the time difference between the onset of arrows and the respective correct or incorrect response, and the posterror slowing (PES) was quantified using the robust measurement method (i.e., the average response time difference between the last correct trial before an error and the first correct trial after an error; [124]).

### 2.5. Electrophysiological Recording and Processing

Continuous EEG data were recorded by 61 equidistant and concentric passive Ag/AgCl-electrodes (Easycap, Herrsching, Germany) and two 32-channel BrainAmp amplifiers (Brain Products GmbH, Gilching, Germany). The recording setup encompassed a band-pass filter of 0.01–250 Hz, a sampling rate of 1000 Hz, a recording reference located between AF3 and Fz, a ground electrode located between AF4 and Fz, and an external electrode placed at the left infraorbital site. During preparation of the electrodes as well as throughout the study, impedances were reduced below a threshold of 5 k*Ω*.

After the recording of the EEG data, they were processed using Brain Vision Analyzer (Brain Products GmbH, Gilching, Germany). In a first step, we administered a band-pass filter with a low cut-off of 0.1 Hz and a high cut-off of 30 Hz (24 dB/oct roll-off) as well as a notch filter of 50 Hz. In a second step, ocular artifacts were corrected by a semi-automatic independent component analysis (ICA; [125]). This procedure involved the automatic identification of statistically independent components of the EEG signal, the visual inspection of each component's scalp topography and component activation, based on which we qualified those components representing ocular artifacts, and the final inspection of the corrected EEG signal. Subsequent to the ocular correction, continuous data of each electrode were re-referenced to the common average reference of all scalp electrodes, which was followed by the segmentation of response-locked epochs (−500 to 800 ms) and the artifact rejection. Segments containing an absolute voltage range exceeding 200 μV, voltage step exceeding 50 μV between consecutive data points, or maximum voltage difference of less than 0.5 μV within 100 ms intervals were removed before averaging each participant's correct and incorrect response segments separately. On average, data loss due to the artifact rejection was small (*M* = 0.87%, *SD* = 1.82%, Max. = 11.90%) in the finally analyzed sample. However, for three participants, data loss was more than 25%, and they were therefore excluded from data analysis ensuring high data quality as is recommended [126]. All participants fulfilled the criterion of at least six erroneous trials to determine a reliable ERN [127].

In accordance with recent guidelines for the quantification of the ERN and CRN in anxiety research [128], we corrected segments for the baseline interval of −500 to −300 ms and scored both ERPs as mean amplitudes between 0 and 100 ms post response at electrode FCz, where the signal was maximal after inspecting the grand-averaged waveforms and topographical distributions. Spearman-Brown corrected correlations of odd- and even-numbered trials indicate good psychometric properties for both the ERN (*r* = 0.94) and CRN (*r* = 0.98).

However, in order to evaluate the impact of different EEG preprocessing decisions on the study results [128–130], we also refer to the multiverse analyses of other commonly used baseline corrections and scoring strategies in the Supporting Informations in detail and report the consistency across different alternative analysis paths including the averaged effect size, as well as standard devation and *p*-range of these effect sizes.

### 2.6. Data Analysis

Data analysis was conducted using IBM SPSS Statistics, version 29.0 (SPSS, Inc., Chicago) with a significance level of *α* = 0.05. Demographic and clinical data including gender, age, education, as well as the aforementioned questionnaires were used to describe our sample. Differences in gender between groups were tested using a *χ*^2^-test. Differences in demographic (age, education), clinical (questionnaires), and behavioral data (accuracy, PES) between the four diagnostic groups were tested by one-way analyses of variance (ANOVAs) with group (OCD, SAD, PHOB, CON) as between-subject factor. Hypotheses on ERPs (ERN and CRN) and behavioral data (response times for correct and incorrect response) were analyzed using separate 2 × 4 mixed-measures ANOVAs with response type (correct, incorrect) as within-subject factor and group (OCD, SAD, PHOB, CON) as between-subject factor. Similar analyses were conducted for the influence of clinical status of an internalizing disorder and family risk for internalizing psychopathology, whereby the between-subject factor group was replaced by clinical status (nonclinical, clinical) and family risk (no risk, risk). Whenever preceding behavioral analyses revealed significant differences, the ERP analyses were controlled for the respective variables. Therefore, we slightly deviated from our preregistration by conducting analyses-of-covariance (ANCOVAs). Follow-up analyses were conducted when results revealed significant interactions. All *p*-values were Sidak-corrected for post hoc comparisons with more than two factor levels.

In order to investigate the potential role of clinical status and the transdiagnostic anxiety dimensions, the sample of the current study was combined with another, subclinical sample [95] that used identical task and outcome specifications. Using multiple linear regression models (separate for ERN and CRN), models included the predictors gender, age, clinical status, PSWQ, and MASQ-AA as well as the interaction terms clinical status × PSWQ, clinical status × MASQ-AA, PSWQ × MASQ-AA, and clinical status × PSWQ × MASQ-AA. We controlled for the potential effects of gender [79, 131, 132] and age [133–135] since previous showed interindividual differences of these demographic variables. Lastly, we were interested in the moderating role of gender on the ERN-PSWQ link. Therefore, we performed multiple linear regression models (separate for ERN and CRN) with the predictors gender, age, clinical status, PSWQ, gender × clinical, and gender × PSWQ. Continuous variables were mean-centered before they were entered in the regression models in order to reduce multicollinearity due to the interaction terms.

## 3. Results

### 3.1. Demographic, Questionnaire, and Clinical Data

#### 3.1.1. Diagnostic Groups

**Figure 1:**
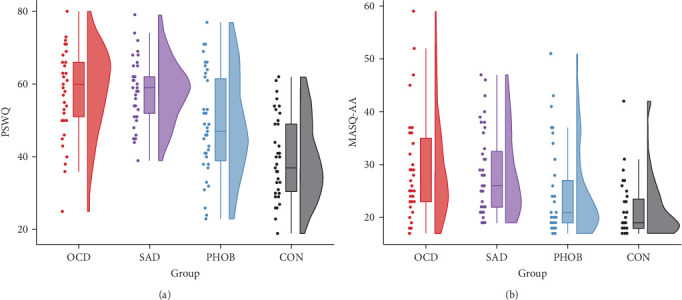
Distribution of anxious apprehension (A) and anxious arousal (B) across diagnostic groups. *Note*. OCD, Obsessive–Compulsive Disorder; SAD, Social Anxiety Disorder; PHOB, Specific Phobia; CON, Control Group; PSWQ, Penn State Worry Questionnaire; MASQ-AA, Mood and Anxiety Symptom Questionnaire (Anxious Arousal Subscale). *N* = 156.

The four groups showed no significant differences regarding gender, age, or years of education. However, as expected, there were significant variations in questionnaire and clinical data. Specific information and statistics for each group are detailed in Table 1. In alignment with the recruitment strategy, obsessive–compulsive symptoms were most prominent in the OCD group, while social anxiety predominated the SAD group. Phobic symptoms also peaked in the PHOB group, but were not significantly different from the OCD and SAD group. Regarding the transdiagnostic anxiety dimensions, anxious apprehension and anxious arousal were highest in the OCD and SAD group, on intermediate levels in the PHOB group, and lowest in the CON group (Figure 1). State and trait anxiety, as well as depressive symptoms, were most pronounced in the OCD and SAD groups, with the CON group displaying the smallest manifestations, and the PHOB group having intermediate symptom severity. Levels of alcohol consumption did not differ significantly between the groups. Overall, the severity of illness in the clinical global impression rating was most severe for the OCD and SAD groups, followed by the PHOB group, and was inconspicuous for the CON group, which was also reflected in the use of antidepressant medication. A similar pattern emerged for global functioning, which was notably impaired in the OCD and SAD groups, moderately reduced in the PHOB group and exhibited its highest level of functioning in the CON group. In summary, the four groups revealed varying profiles concerning the symptom severity and the anxiety dimensions. Regarding the severity of illness, use of antidepressant medication, and functional impairment, the OCD and SAD groups were more affected than the PHOB and CON groups.

#### 3.1.2. Clinical Status and Family Risk

Since we also grouped participants in terms of clinical status of an internalizing disorder and family risk for internalizing psychopathology, we report group-specific means, standard deviations, and statistical results of the group comparison in the Supporting Information (Table S2). Across groups, symptoms displayed a spectrum of severity, with clinical participants with family risk showing the most severe symptoms, succeeded by clinical participants without family risk, nonclinical participants with family risk, and nonclinical participants without family risk.

### 3.2. Behavioral Data

#### 3.2.1. Diagnostic Groups

The categorical analyses of the diagnostic groups did not reveal any significant differences regarding accuracy, *F* (3, 152) = 0.16, *p*=0.923, *η*_*p*_^2^ = 0.00, or PES, *F* (3, 152) = 0.69, *p*=0.558, *η*_*p*_^2^ = 0.01. Overall response times were faster on error compared with correct trials across groups, *F* (1, 152) = 522.81, *p* < 0.001, *η*_*p*_^2^ = 0.78, but did not differ between groups, *F* (3, 152) = 1.70, *p*=0.169, *η*_*p*_^2^ = 0.03. However, we found an interaction effect of response × group on response times, *F* (3, 152) = 5.40, *p*=0.001, *η*_*p*_^2^ = 0.10. Post-hoc comparisons showed that the PHOB group responded faster on error trials than the CON group (*p*=0.031), with no other difference passing the significance threshold (all *p*s > 0.48). Group-specific means and standard deviations of the behavioral data can be found in Table 2. In brief, behavioral performance did not differ significantly between the four diagnostic groups, with the exception of error response times, whereby the PHOB group demonstrated faster responses than the CON group.

#### 3.2.2. Clinical Status and Family Risk

Since we also investigated the influence of clinical status of an internalizing disorder and family risk for internalizing psychopathology on ERPs, we shortly report results of participants' behavioral performance here. Regarding accuracy, we did not observe a significant effect of clinical status, *F* (1, 152) = 0.42, *p*=0.516, *η*_*p*_^2^ = 0.00, family risk, *F* (1, 152) = 2.05, *p*=0.155, *η*_*p*_^2^ = 0.01, or clinical status × family risk, *F* (1, 152) = 0.01, *p*=0.915, *η*_*p*_^2^ = 0.00. However, PES differed as a result of family risk, *F* (1, 152) = 6.94, *p*=0.009, *η*_*p*_^2^ = 0.04, with prolonged PES for the risk group, while there was no significant effect of clinical status, *F* (1, 152) = 0.27, *p*=0.607, *η*_*p*_^2^ = 0.00, or clinical status × family risk, *F* (1, 152) = 2.28, *p*=0.133, *η*_*p*_^2^ = 0.02. Overall response times were faster on error compared with correct trials, *F* (1, 152) = 269.51, *p* < 0.001, *η*_*p*_^2^ = 0.64. In addition, there was a significant interaction effect of response × clinical status on response times, *F* (1, 152) = 5.36, *p*=0.022, *η*_*p*_^2^ = 0.03, which was qualified by faster response times for error trials (*p*=0.087) but not for correct trials (*p*=0.682) for the clinical compared with the nonclinical group, albeit closely missing the significance threshold for error trials in the post hoc comparison. No other significant effects on response times were found (all *p*s > 0.11). Group-specific means and standard deviations of the behavioral data can be found in the Supporting Information (Table S3). Taken together, family risk was associated with prolonged PES and, on a trend level, clinical status with faster error response times.

### 3.3. Event-Related Potentials

#### 3.3.1. Diagnostic Groups

**Figure 2:**
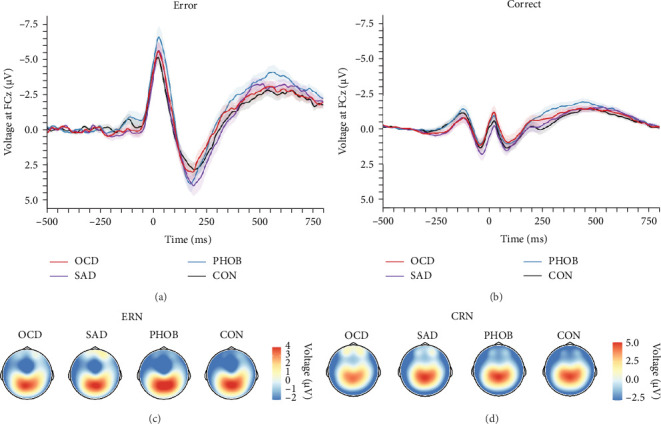
Grand-Averaged Waveforms and Corresponding Topographies for Error and Correct Trials of the Diagnostic Groups. *Note*. Response-locked grand-averaged waveforms of erroneous and correct trials of each group (A, B). OCD, obsessive–compulsive disorder; SAD = social anxiety disorder; PHOB, specific phobia; CON, control group. Corresponding topographic head maps of the ERN (C) and CRN (D). CRN, correct-response negativity; ERN, error-related negativity. *N* = 156.

In the categorical analysis of the four diagnostic groups, we slightly deviated from our preregistered model (ANOVA) by controlling for error response times (ANCOVA), given that the PHOB group showed faster error response times compared with the CON group. The ANCOVA revealed a main effect of response type indicating larger ERN compared with CRN amplitudes, *F* (1, 151) = 19.89, *p* < 0.001, *η*_*p*_^2^ = 0.12. However, we did not find a significant group effect, *F* (3, 151) = 1.56, *p*=0.201, *η*_*p*_^2^ = 0.03, nor an interaction of response × group, *F* (3, 151) = 0.95, *p*=0.418, *η*_*p*_^2^ = 0.02, meaning we could not find evidence for differences in ERN or CRN amplitudes between the diagnostic groups. Table 2 lists group-specific means and standard deviations of the ERN and CRN.  Figure 2 shows grand-averaged potentials and topographies of each group. This result pattern was robust against different quantification approaches of the ERN and CRN (Table S4 in the Supporting Information). Across scoring strategies, the average effect size was *M*_*η*_*p*_^2^_ = 0.114, *SD*_*η*_*p*_^2^_ = 0.026 (*p*-range = < 0.001–0.001) for the response effect, *M*_*η*_*p*_^2^_ = 0.020, *SD*_*η*_*p*_^2^_ = 0.007 (*p*-range = 0.244–0.696) for the group effect, and *M*_*η*_*p*_^2^_ = 0.021, *SD*_*η*_*p*_^2^_ = 0.004 (*p*-range = 0.242–0.479) for the response × group interaction.

In order to examine the influence of our naturalistic control group on ERP differences, we repeated the categorical analyses by excluding five participants with a current or lifetime internalizing diagnosis from our control group. In this ANCOVA involving a strictly healthy control group, the group factor narrowly missed the significance threshold, *F* (3, 146) = 2.49, *p*=0.062, *η*_*p*_^2^ = 0.05. Consequently, post hoc comparisons also fell short of significance, although the comparison related to ERN differences between PHOB group (*M* = −4.64, *SD* = 4.22) and HC group (*M* = −2.58, *SD* = 2.68) approached the threshold (*p*=0.070). Additionally, there was a main effect of response type, *F* (1, 146) = 18.05, *p* < 0.001, *η*_*p*_^2^ = 0.11, but no interaction effect of response × group, *F* (3, 146) = 1.03, *p*=0.379, *η*_*p*_^2^ = 0.02. Grand-averaged potentials of the three clinical groups compared with the healthy control group can be found in Figure S2 of the Supporting Informations. Across scoring strategies, the average effect size was *M*_*η*_*p*_^2^_ = 0.109, *SD*_*η*_*p*_^2^_ = 0.025 (*p*-range = < 0.001–0.002) for the response effect, *M*_*η*_*p*_^2^_ = 0.046, *SD*_*η*_*p*_^2^_ = 0.010 (*p*-range = 0.033–0.265) for the group effect, and *M*_*η*_*p*_^2^_ = 0.017, *SD*_*η*_*p*_^2^_ = 0.002 (*p*-range = 0.379–0.520) for the response × group interaction. For more statistical details, please see Table S5 in the Supporting Informations. Finally, the pattern of results did not change when excluding participants using antidepressant medication, still not revealing significant effects of the group factor, *F* (3, 136) = 1.31, *p*=0.273, *η*_*p*_^2^ = 0.03, nor response × group factor, *F* (3, 136) = 0.42, *p*=0.741, *η*_*p*_^2^ = 0.01. We also investigated the potential relationship of the ERN and CRN with global symptom measures, but neither severity of illness (CGI-S: *r*_ERN_ = -0.05, *p*=0.538; *r*_CRN_ = -0.10, *p*=0.237) nor global functioning (GAF: *r*_ERN_ = 0.07, *p*=0.382; *r*_CRN_ = 0.12, *p*=0.150) showed significant correlations.

In essence, our investigation did not reveal evidence for variations in ERN or CRN amplitudes among the clinical groups, nor in comparison to the naturalistic control group. However, upon refining the control group by excluding participants with a current or lifetime internalizing disorder, creating a more strictly defined healthy control group, trend-level significant differences emerged between the PHOB and HC group.

#### 3.3.2. Clinical Status and Family Risk

**Figure 3:**
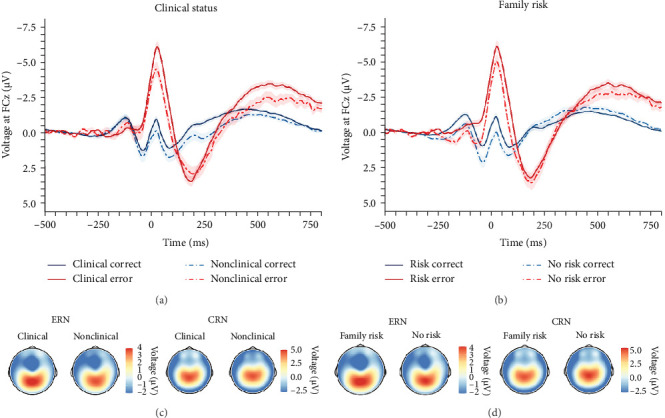
Grand-averaged waveforms and corresponding topographies of error and correct trials for participants with or without a clinical status of an internalizing disorder or family risk for internalizing psychopathology. *Note*. Response-locked grand-averaged waveforms of correct and erroneous trials for participants with or without a clinical status of an internalizing disorder (A) as well as for participants with or without a family risk for internalizing psychopathology (B). Corresponding topographic head maps of the ERN and CRN (C, D). ERN, error-related negativity; CRN, correct-response negativity. *N* = 156.

In order to investigate the role of clinical status of an internalizing disorder and family risk for internalizing psychopathology, we conducted a nonpreregistered categorical analysis (ANCOVA) after reclassifying participants (i.e., clinical/nonclinical participants with/without family risk), while controlling for PES and error response times (see behavioral data for details). Detailed results of the ANCOVA are listed in Table 3 and illustrated in Figure 3. We found a main effect of response with larger ERN compared with CRN, but clinical status closely missed the significance threshold (*p*=0.071), although there were descriptive differences in both ERPs, with larger ERN (nonclinical: *M* = −2.58, *SD* = 2.68 vs., clinical: *M* = −4.04, *SD* = 3.69) as well as CRN amplitudes (nonclinical: *M* = 0.91, *SD* = 2.27 vs., clinical: *M* = 0.21, *SD* = 2.48). Instead, family risk was associated with higher ERN (no risk: *M* = −2.79, *SD* = 3.00 versus risk: *M* = −4.21, *SD* = 3.71) and CRN amplitudes (no risk: *M* = 0.94, *SD* = 2.02 versus risk: *M* = 0.06, *SD* = 2.60). Notably, the result pattern for the family risk effect was heterogeneous across scoring strategies, while the clinical effect was significant across all other scoring strategies except for the preregistered one reported here (Table S6 in the Supporting Information): The average effect size was *M*_*η*_*p*_^2^_ = 0.035, *SD*_*η*_*p*_^2^_ = 0.012 (*p*-range = 0.004–0.071) for the clinical effect, *M*_*η*_*p*_^2^_ = 0.014, *SD*_*η*_*p*_^2^_ = 0.011 (*p*-range = 0.039–0.861) for the family risk effect, and *M*_*η*_*p*_^2^_ = 0.000, *SD*_*η*_*p*_^2^_ = 0.000 (*p*-range = 0.652–0.902) for the clinical × family risk interaction. In summary, both clinical status of an internalizing disorder and family risk for internalizing psychopathology seem to be associated with an enhanced ERN as well as CRN, although statistical significance of these effects were partially depending on the chosen scoring strategy.

#### 3.3.3. Clinical Status and Anxiety Dimensions

In accordance with our preregistration, we also combined data of the present clinical study with data from a previously published subclinical study [95] to investigate the impact of clinical status as well as the role of transdiagnostic anxiety dimensions across a wider spectrum of symptom severity. This yielded a joint sample consisting of 246 individuals affected by at least one lifetime internalizing disorder (*n* = 136) and individuals without any lifetime history of psychopathology (*n* = 110). Unfortunately, data concerning family risk for internalizing psychopathology were not collected in the previous study and could therefore not be analyzed in this larger, combined sample. Since clinical status, PSWQ, and MASQ-AA were our predictors of main interest, we will focus on these in the following description of results. However, detailed results of the linear regression models of the ERN and CRN including all variables are listed in Table 4. Results of different scoring strategies are summarized in the Supporting Information (Table S7).

**Figure 4:**
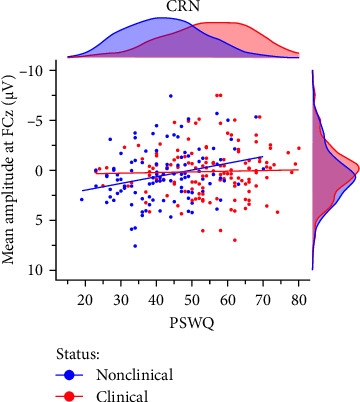
Clinical status moderating the link between anxious apprehension and CRN. *Note*. CRN, correct-response negativity. PSWQ, Penn State Worry Questionnaire. *N* = 246.

In the preregistered ERN model, clinical status closely missed the significance threshold (*p*=0.069) but was significant for all other scoring strategies. The average effect size of clinical status was *M*_*β*_ = −0.18, *SD*_*β*_ = 0.04 (*p*-range = 0.003– 0.069) across scoring strategies. No other predictor of main interest (i.e., PSWQ or MASQ-AA) was significant. In the preregistered CRN model, we found a significant interaction of clinical status × PSWQ (*p*=0.032; Figure 4) which was qualified by larger CRN amplitudes with increasing anxious apprehension in nonclinical (*r* = −0.27, *p*=0.005) but not clinical participants (*r* = −0.03, *p*=0.722). Across scoring strategies, the average effect size of clinical × PSWQ was *M*_*β*_ = 0.31, *SD*_*β*_ = 0.04 (*p*-range = 0.004– 0.040). The average Pearson correlation between PSWQ and the CRN across scoring strategies was *M*_*r*_ = −0.26, *SD*_*r*_ = 0.04 (*p*-range = < 0.001–0.054) in nonclinical participants and *M*_*r*_ = −0.01, *SD*_*r*_ = 0.05 (*p*-range = 0.372–0.958) in clinical participants. Consequently, depending on the scoring approach, larger ERN amplitudes were linked to clinical status mirroring the effects observed in the categorical analysis. For the CRN, we found a differential effect regarding the PSWQ-CRN link between nonclinical and clinical participants, such that only nonclinical participants showed larger CRN amplitudes with increasing anxious apprehension.

As preregistered, we also investigated the role of the transdiagnostic anxiety dimensions within the initial sample (*n* = 156). Detailed results of these models (with smaller statistical power) can be found in the Supporting Information (Table S8). For the ERN, we found partial support of an association with PSWQ, depending on the scoring strategy, with an average effect size of *M*_*β*_ = −0.17, *SD*_*β*_ = 0.03 (*p*-range = 0.044 –0.248). No association was found for the MASQ-AA (all *p*s > 0.26). For the CRN, we could not find any significant prediction by PSWQ or MASQ-AA (all *p*s > 0.16).

#### 3.3.4. Anxious Apprehension and Gender

**Figure 5:**
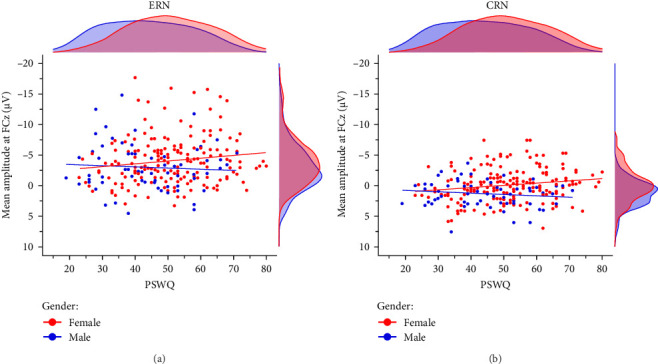
Gender moderating the link bbetween anxious apprehension and (A) ERN as Well as (B) CRN. *Note*. CRN, correct-response negativity; ERN, error-related negativity; PSWQ, Penn State Worry Questionnaire. *N* = 246.

Prior research has indicated that gender may act as a moderating factor in the relationship between anxiety and enhanced error monitoring, with women displaying a stronger association between anxious apprehension and increased ERN amplitudes [94]. In order to investigate the moderating role of gender, we conducted explorative multiple linear regression models using the combined sample (*n* = 246) including 181 women and 65 men. The results demonstrated that the interaction term gender × PSWQ was significant for both the ERN and CRN indicating a differential effect, whereby only women exhibited a larger ERN (females: *r* = −0.15, *p*=0.049 vs., males: *r* = 0.07, *p*=0.584) and CRN (females: *r* = −0.17,*p*=0.023 vs., males: *r* = 0.14, *p*=0.261) with increasing anxious apprehension (Table 5, Figure 5). Detailed results across various scoring strategies are available in the Supporting Informations (Table S9). Across scoring strategies, the average effect size of gender × PSWQ was *M*_*β*_ = 0.61, *SD*_*β*_ = 0.10 (*p*-range = 0.002–0.035) for the ERN and *M*_*β*_ = 0.60, *SD*_*β*_ = 0.04 (*p*-range = 0.005– 0.016) for the CRN. The average Pearson correlation between PSWQ and the ERN in women was *M*_*r*_ = -0.16, *SD*_*r*_ = 0.02 (*p*-range = 0.016−0.071) and *M*_*r*_ = 0.13, *SD*_*r*_ = 0.04 (*p*-range = 0.139–0.584) in men. For the PSWQ-CRN link, the average correlation was *M*_*r*_ = −0.15, *SD*_*r*_ = 0.02 (*p*-range = 0.015–0.104) for women and *M*_*r*_ = 0.13, *SD*_*r*_ = 0.04 (*p*-range = 0.160–0.530) for men. Taken together, women but not men showed increasing ERN and CRN amplitudes with increasing anxious apprehension.

## 4. Discussion

This preregistered study examined the influence of disorder category, clinical status, family risk, and transdiagnostic symptom dimensions on the ERN and CRN in a large sample of patients with OCD, SAD, specific phobia, and a naturalistic control group. Results did not reveal significant differences in ERN or CRN among the clinical groups (i.e., OCD, SAD, and specific phobia), nor in comparison to the naturalistic control group. However, after creating a more strictly defined healthy control group, we found larger ERN amplitudes in the specific phobia compared with the healthy control group. In addition, when comparing clinical participants with a lifetime history of an internalizing disorder to those without any past or present diagnoses, both ERN and CRN were larger in the clinical group. Moreover, our data also suggest an association of increased ERN and CRN amplitudes with family risk for internalizing psychopathology. Lastly, the dimensional analyses did not reveal an overall association between anxious apprehension and the ERN or CRN, respectively. Instead, we could identify gender as a moderating factor in this relationship, indicating that higher anxious apprehension was associated with larger ERN and CRN amplitudes in women but not in men.

In a broader context, our disorder group-based analyses contradict findings reported in the literature (e.g., [56, 75]), as we do not replicate the expected group differences between the control group and OCD or SAD, respectively. Furthermore, contrary to our expectations, we observed trend level significantly greater ERN amplitudes in the specific phobia group when compared to a strictly defined healthy control group, which has not yet been suggested by previous research [85, 86]. However, to the best of our knowledge, our study is the first to include a clinically phobic group that is also larger than those in previous studies. Consequently, our findings may contribute to the current understanding of neural error monitoring in these disorders. While an augmented ERN is commonly conceptualized as the sensitivity to internal threat (i.e., concerns about the consequences of one's performance and potentially the social evaluation by others) rather than external threats (i.e., fear of specific objects or situations) and thereby linked to disorders, such as OCD, SAD, and GAD, it might also be adaptive for individuals with fear-related disorders, such as specific phobia or agoraphobia, to monitor their behavior carefully.

However, it is crucial to consider the possibility that these findings may, in part, be attributed to insufficient statistical power, as the disorder-specific analysis was only able to detect medium or large-sized effects. By reclassifying all clinical participants within one group and combining data with a previous subclinical study, we were able to conduct better-powered analyses revealing larger ERN and CRN amplitudes in the clinical group. Consistent with prior investigations, our results undermine the importance of clinical status [53], particularly the role of lifetime internalizing disorders [95], for the link between anxiety and enhanced performance monitoring. Individuals with internalizing psychopathology seem to share a predisposition for a cognitive system that is more wired to monitor performance compared with those without any clinically relevant symptomatology.

In addition to clinical status, family risk for internalizing psychopathology may also help us understand the role of neural performance monitoring in the development of anxiety and obsessive–compulsive symptoms. A limitation inherent to cross-sectional studies, including our own, lies in the inability to establish causal relationships between enhanced performance monitoring and the development of symptoms or vice versa. Nonetheless, at-risk participants can help identify potential biomarkers or patterns associated with at-risk status rather than current symptoms that may precede the onset of disorders. Following this idea, our results showing increased ERN and CRN amplitudes in participants with familial risk contribute to the notion that enhanced ERN and CRN amplitudes serve as vulnerability markers, that may be transmitted within families, indicating a predisposition toward internalizing disorders [48, 64, 67]. Clinical status and family risk did not yield an additive effect on the magnitude of performance monitoring; so either factor independently proved sufficient to be associated with heightened ERN and CRN amplitudes. In summary, enhanced performance monitoring might contribute to explain the trajectories to clinical anxiety and obsessive–compulsive symptoms [36, 136] and an elevated ERN may serve as a broad risk marker for the anxiety and obsessive–compulsive spectrum, rather than being indicative for specific disorders within that spectrum.

In this context, it is crucial to differentiate between the trajectories leading to an enhanced trait-like ERN and its role in the etiopathogenesis of clinical anxiety and obsessive–compulsive symptoms. On the one hand, an increased ERN is potentially shaped by a complex interplay of multiple early-acting factors during childhood and adolescence, such as genetics [72, 73], gender [131, 132, 137, 138], cognitive control [139], temperament [140–143], and parenting style [73, 140, 144–146]. On the other hand, an increased ERN represents a risk marker for anxiety and obsessive–compulsive disorders, regardless of the specific etiological factors behind its enhancement. Consequently, while some individuals may have inherited an enhanced ERN, posing a risk for clinical anxiety and obsessive–compulsive symptoms, this aspect may only account for the phenomenon in a subset of cases. In essence, an individual exposed to a punitive parenting style that results in an enhanced ERN may harbor a comparable risk for clinical symptoms as individuals who have inherited an elevated ERN. Yet, the complex interplay of these influencing factors contributing to an elevated trait-like ERN remains widely unexplored. Consequently, future investigations should prioritize a comprehensive examination of their combined effects to gain deeper insights into the genesis of enhanced error-related brain activity. In addition, these different trajectories and combinations of etiological factors could also serve as a starting point for patient stratification and contribute to the establishment of precision medicine approaches.

Another potential explanation for previously observed differential associations within the anxiety and obsessive–compulsive spectrum highlights the pivotal role of the transdiagnostic dimension anxious apprehension [52, 53]. An enhanced ERN does not seem to generally indicate risk for all disorder categories of the anxiety and obsessive–compulsive spectrum [85, 86, 147]; instead, enhanced error monitoring likely contributes only to the development of disorders characterized by anxious apprehension [141, 147, 148]. However, an important remark to consider is that the relationship between elevated error monitoring and anxious apprehension is, to a significant extent, relying on the retrospective classification of samples (such as worry versus mixed anxiety) in meta-analyses. Consequently, the direct dimensional association between ERN amplitudes and anxious apprehension vs. anxious arousal has received comparatively limited attention, with only some exceptions (e.g., [79, 90, 92, 95]). To the best of our knowledge, our study is among the first to employ a transdiagnostic dimensional approach within a widely transdiagnostic dataset including different clinical disorder categories.

Aligning with the findings of previous research, our results support the notion that the link between heightened anxious apprehension and an enhanced ERN is gender-specific, such that it predominantly manifests in women [94]. Extending previous research findings, we found evidence that the gender-specific link of anxious apprehension also encompasses the CRN. Overall, these findings might imply differential trajectories of anxiety in women and men, wherein an enhanced ERN and CRN appear to only be associated with anxious apprehension among women, not men. Within the existing literature, two explanatory approaches have been advanced to elucidate the gender-specific link between anxious apprehension and increased error monitoring [94].

One perspective posits that the amplification of verbal worries exerts a more pronounced impact on cognitive performance in women. Extensive research demonstrates that anxiety is cross-sectionally and prospectively linked to impairments in cognitive functioning [149–151] with some studies indicating greater impairment in women [152–154]. This may be attributable to the inclination of women, relative to men, to employ verbal processes during problem-solving, thereby fostering subvocal articulation as a response tendency to threatening or uncertain situations [94]. Additionally, the functional connectivity of fronto-temporo-parietal brain areas in interoception seems to be more pronounced in females [155] which may suggest that women may have a more integrated neural network for processing internal bodily signals. Alternatively, another perspective underscores the organizational and regulatory impact of ovarian hormones on frontal brain regions associated with cognitive control in women [156–158]. Moser et al. [94] argue that the elevated concentration of estradiol in regions linked to error monitoring, coupled with estradiol's modulatory influence on dopamine—a primary neurotransmitter thought to contribute to the generation of the ERN—may offer a promising neurobiological rationale for the observed connection between anxious apprehension and the ERN in women. A recent study suggests that estradiol and progesterone may represent protective factors, wherein women exhibiting relatively elevated levels of these hormones show a weaker association of worry and error-related brain activity in the ACC [157]. While our study design cannot offer support for either explanation regarding the gender specificity of this connection, we assert that it is imperative to consider gender effects when investigating the etiological pathways of the neural foundations in clinical anxiety.

However, even though our study found that anxious apprehension was dimensionally linked to enhanced error monitoring (in women), this did not directly translate into ERN differences among the investigated clinical groups. This challenges the assumption that variations in latent symptom profiles, specifically anxious apprehension, underlying anxiety and obsessive–compulsive disorders can singularly account for driving this link. It is plausible that another transdiagnostic and more general phenotype, such as threat sensitivity [159], trait defense reactivity [4], or anxious misery [160], that is on the one hand partially associated with anxious apprehension but potentially more evenly distributed across internalizing disorder categories, might drive variations in ERN amplitudes, thus, explaining that we did not find ERN differences between disorder categories. Nevertheless, examining how dimensional constructs translate into differences between disorder categories requires future well-powered studies involving multiple disorders categories with larger samples sizes for each group.

In evaluating our results, it is essential to consider certain limitations. Firstly, we did not include the full range of potentially relevant disorders of the anxiety and obsessive–compulsive spectrum. Of particular interest would be GAD, panic disorder, and agoraphobia in order to cover the complete transdiagnostic spectrum. Furthermore, regarding the control participants, we followed a more conservative approach by choosing a naturalistic control group. In a similar vein, we included participants who were currently using medication. However, the ERN is known to be potentially influenced by medication, particularly antipsychotic drugs that affect the dopaminergic system [161–163]. In our study, only a subset of participants (*n* = 15) were using psychopharmacological medication (i.e., antidepressant drugs) to treat their psychiatric conditions. However, control analyses did not indicate significant differences in ERPs. Moreover, participants who had used any form of neuroleptic medication within the past three months or any form of benzodiazepines within the last four weeks were excluded from the study to mitigate the potential confounding effects of psychopharmacological drug use. Nonetheless, there remains a possibility that antidepressant medication use of a subset of our sample could still affect the presented results.

Another limitation is that we utilized a speeded version of the flanker task; initially in order to promote potential group differences between clinical and control participants, as OCD patients previously showed impaired flexibility under speed conditions [39]. However, this pattern was not yet investigated for SAD or specific phobia and it remains unclear whether this inflexibility also applies to these disorders. The speed instructions might have also led to smaller ERN amplitudes [5, 164], possibly limiting the range of variance available to analyze. Lastly, aiming at demonstrating the robustness or sensitivity of the results to different forking paths in ERP quantification, some few of the analyses (e.g., family risk) displayed substantial variation in the final outcome. Over the past years of anxiety research, the potential influence of methodological decisions in EEG preprocessing on study results has received increasing attention [128–130]. However, final guidelines that stand on a clear and unequivocal foundation of empirical evidence are yet to be formulated [165].

Finally, we want to emphasize the importance of further investigating the mechanisms involved in translating neural indices of vulnerability into clinical anxiety and its diverse facets. Previous research (e.g., [136, 166]) has frequently invoked the diathesis-stress model, showing that instances of adverse life events, such as interpersonal stress [167], a natural disaster [168], or a pandemic [169] appear to moderate the association between enhanced neural error monitoring and the development of anxiety and obsessive–compulsive symptoms. Moreover, there is evidence that not only stressful life events (i.e., environmental factors) serve as moderators but also other features of a person (i.e., individual factors), such as a behavioral inhibition [141, 142] or biological sex [94], in conjunction with enhanced error monitoring, play a role in the emergence of clinical anxiety. In light of these observations, we believe that the research field would notably benefit from the formulation of a more detailed and integrative model delineating the trajectories from altered neural error monitoring to psychopathological conditions. In our opinion, this model should consider (a) the general formation of an increased or decreased trait-like ERN by various early-acting factors (e.g., genetics, cognitive control, or parenting style), (b) the interplay of an enhanced or diminished trait-like ERN with environmental (e.g., adverse life events) and individual factors (e.g., biological sex or behavioral inhibition) resulting in either internalizing or externalizing disorders, and (c) the characterization of these disorders by specific facets (e.g., impulsivity or anxious apprehension).

## 5. Conclusion

In conclusion, this study suggests that increased ERN and CRN amplitudes serve as neural risk makers indicating vulnerability for internalizing psychopathology, particularly across the anxiety- and obsessive–compulsive spectrum. Additionally, our findings highlight the role of clinical status, gender, family risk, and the transdiagnostic anxiety dimension of anxious apprehension. Future research endeavors should aim at unraveling the trajectories leading to internalizing psychopathology by examining the complex interplay between the predisposition for enhanced neural performance monitoring and various biological, psychological, and environmental factors.

## Figures and Tables

**Table 1:** Demographical, questionnaire, and clinical data across diagnostic groups.

Variable	OCD (*n* = 39)		SAD (*n* = 39)		PHOB (*n* = 39)		CON (*n* = 39)		Group comparison			
	*M*	*SD*	*M*	*SD*	*M*	*SD*	*M*	*SD*	*χ* ^2^ */F*	*df*	*η* ^2^	*p*
Demographical												
Gender (*f*/*m*)	28/11	—	29/10	—	30/9	—	28/11	—	0.36	3	—	0.948
Age	28.36	6.38	28.38	7.64	27.90	9.80	29.41	8.31	0.24	3, 152	0.01	0.868
Education	12.23	1.01	12.23	1.11	12.18	1.02	12.18	0.94	0.03	3, 152	0.00	0.992
Questionnaires												
OCI-R	28.31_a_	11.38	11.82_b_	8.79	9.21_bc_	10.11	5.59_c_	4.77	47.49	3, 152	0.48	**<0.001**
LSAS-SR	47.31_a_	30.33	69.74_b_	29.01	31.24_c_	24.15	19.46_c_	14.94	28.71	3, 151	0.36	**<0.001**
SMSP	8.26_a_	8.38	6.56_a_	7.38	8.79_a_	7.86	1.34_b_	2.34	9.14	3, 150	0.16	**<0.001**
PSWQ	58.13_a_	11.75	58.10_a_	8.73	48.56_b_	13.90	39.41_c_	11.13	23.65	3, 152	0.32	**<0.001**
MASQ-AA	29.69_a_	10.12	28.15_ab_	7.91	24.62_bc_	8.20	21.85_c_	5.98	7.26	3, 152	0.13	**<0.001**
STAI-T	52.67_a_	11.42	53.23_a_	10.17	40.08_b_	12.52	34.85_b_	7.49	29.53	3, 152	0.37	**<0.001**
STAI-S	45.28_a_	13.52	46.03_a_	11.03	35.05_b_	9.23	32.42_b_	5.81	17.55	3, 150	0.26	**<0.001**
BDI-II	17.41_a_	11.70	17.08_a_	12.44	8.00_b_	9.62	3.74_b_	4.61	17.77	3, 152	0.26	**<0.001**
AUDIT	4.23	3.74	3.64	2.85	4.51	2.86	3.87	3.11	0.58	3, 152	0.01	0.630
EHI	78.72	31.55	75.64	35.47	65.77	46.16	76.92	29.53	1.00	3, 152	0.02	0.395
Clinical												
Medication (*y*/*n*)	9/39	—	4/35	—	2/37	—	0/39	—	13.20	3	—	**0.004**
Diagnoses	2.90_ab_	1.79	3.00_a_	1.93	2.00_b_	1.52	0.13_c_	0.34	29.47	3, 152	0.37	**<0.001**
CGI-S	3.59_a_	0.85	3.58_a_	1.03	2.79_b_	1.01	1.00_c_	0.61	73.18	3, 151	0.59	**<0.001**
GAF	56.23_a_	9.20	58.61_a_	11.47	75.28_b_	11.53	88.49_c_	6.33	91.78	3, 151	0.65	**<0.001**
Y-BOCS	22.03	5.34	—	—	—	—	—	—	—	—	—	—

*Note*: *p* < 0.05 are printed in bold. Medication (y = yes, n = no) includes psychopharmacological treatment with antidepressants as reported by the participants; Diagnoses include index and comorbid diagnoses (*n*); Gender (f = female, m = male); age and education in years. Degrees of freedom are deviating for some questionnaires due to missing data; means with different subscripts within rows indicate significant differences according to Sidak corrected post-hoc *t*-tests with *p* <0.05. *N* = 156.

Abbreviations: AUDIT, Alcohol Use Disorders Identification Test; BDI-II, Beck Depression Inventory II; CGI-S, Clinical Global Impression – Severity of Illness; CON, control group; EHI, Edinburgh Handedness Inventory (Modified); GAF, Global Assessment of Functioning; LSAS-SR, Liebowitz Social Anxiety Scale – self report; MASQ-AA, Mood and Anxiety Symptom Questionnaire (Anxious Arousal Subscale); OCD, obsessive–compulsive disorder; OCI-R, Obsessive–Compulsive Inventory; PHOB, specific phobia; PSWQ, Penn State Worry Questionnaire; SAD, social anxiety disorder; SMSP, Severity Measure for Specific Phobia; STAI, State-–Trait-Anxiety Inventory (Trait and State Subscale); Y-BOCS, Yale-Brown Obsessive Compulsive Scale.

**Table 2:** Electrophysiological and behavioral data of the diagnostic groups.

Variable	OCD (*n* = 39)		SAD (*n* = 39)		PHOB (*n* = 39)		CON (*n* = 39)	
	*M*	*SD*	*M*	*SD*	*M*	*SD*	*M*	*SD*
ERPs								
ERN (μV)	−3.71	3.42	−3.47	3.48	−4.64	4.22	−3.08	2.85
CRN (μV)	−0.01	2.68	0.78	2.44	0.19	2.22	0.49	2.45
Behavior								
Accuracy (%)	86.07	7.80	85.53	8.54	85.50	7.53	84.74	10.07
RT correct (ms)	431.41	47.67	415.30	46.16	420.62	35.84	430.11	48.84
RT incorrect (ms)	359.70	59.74	359.05	64.67	343.27	39.30	380.45	64.52
PES (ms)	43.70	30.12	43.02	24.71	40.30	22.52	35.88	28.54

*Note: N* = 156.

Abbreviations: CON, control group; CRN, correct-response negativity; ERN, error-related negativity; ERPs, event-related potentials; OCD, obsessive–compulsive disorder; PES, post-error slowing; PHOB, specific phobia; RT, response time; SAD, social anxiety disorder.

**Table 3:** The effects of clinical status of an internalizing disorder and family risk for internalizing psychopathology on the ERN and CRN.

Variable	*F*	*df*	*η*_*p*_^2^	*p*
Response	19.10	1, 150	0.11	**<0.001**
Response × Clinical	0.69	1, 150	0.01	0.406
Response × FHS Internalizing	0.54	1, 150	0.00	0.466
Response × Clinical × FHS Internalizing	0.03	1, 150	0.00	0.860
Clinical	3.32	1, 150	0.02	0.071
Clinical × FHS Internalizing	0.12	1, 150	0.00	0.731
FHS Internalizing	4.35	1, 150	0.03	**0.039**

*Note:* Analyses were controlled for error response time and post-error slowing, as these behavioral variables differed between the respective groups. *N* = 156. *p* < 0.05 are printed in bold. clinical (nonclinical, clinical); response (correct, incorrect).

Abbreviations: CRN, correct-response negativity; ERN, error-related negativity; FHS, Family History Screen (family risk, no family risk).

**Table 4:** The role of clinical status, PSWQ, and MASQ-AA on the ERN and CRN within the combined sample across the severity continuum.

Variable	ERN									CRN								
	*b*	*SE*_*boot*_	*β*	*t*	*p*_*boot*._	*R* ^2^_*corr*_	*F*	*df*	*p*	*b*	*SE*_*boot*_	*β*	*t*	*p*_*boot*._	*R* ^2^_*corr*_	*F*	*df*	*p*
Model						0.02	1.62	9, 236	0.110						0.08	3.35	9, 236	**<0.001**
Gender	0.78	0.61	0.09	1.33	0.198					1.08	0.34	0.18	2.85	**0.002**				
Age	0.07	0.03	0.13	1.97	**0.017**					0.02	0.02	0.06	0.87	0.320				
Clinical	−1.07	0.59	−0.14	−1.70	0.069					0.05	0.41	0.01	0.13	0.907				
PSWQ	−0.04	0.05	−0.13	−0.89	0.366					−0.06	0.03	−0.28	−1.90	0.060				
MASQ-AA	0.03	0.06	0.07	0.48	0.569					−0.01	0.04	−0.04	−0.31	0.766				
Clinical × PSWQ	0.04	0.06	0.10	0.71	0.476					0.08	0.04	0.27	2.12	**0.032**				
Clinical × MASQ-AA	−0.09	0.08	−0.17	−1.16	0.244					−0.02	0.05	−0.06	−0.42	0.668				
PSWQ × MASQ-AA	−0.01	0.01	−0.20	−1.36	0.294					0.00	0.00	−0.15	−1.04	0.351				
Clinical × PSWQ × MASQ-AA	0.01	0.01	0.24	1.54	0.194					0.00	0.00	0.01	0.05	0.969				

*Note*: *p* < 0.05 are printed in bold. Gender (0 = female, 1 = male); age in years; clinical (0 = nonclinical, 1 = clinical). Continuous variables were mean-centered. *N* = 246.

Abbreviations: CRN, correct-response negativity; ERN, error-related negativity; MASQ-AA, Mood and Anxiety Symptom Questionnaire; PSWQ, Penn State Worry Questionnaire.

**Table 5:** The role of gender and PSWQ on the ERN and CRN within the combined sample across the severity continuum.

Variable	ERN									CRN								
	*b*	*SE*_*boot*_	*β*	*t*	*p*_*boot*._	*R* ^2^_*corr*_	*F*	*df*	*p*	*b*	*SE*_*boot*_	*β*	*t*	*p*_*boot*._	*R* ^2^_*corr*_	*F*	*df*	*p*
Model	—	—	—	—	—	0.03	2.41	6, 239	**0.028**	—	—	—	—	—	0.06	3.79	6, 239	**0.001**
Gender	1.30	0.93	0.15	1.34	0.155	—	—	—	—	1.76	0.63	0.30	2.78	**0.002**	—	—	—	—
Age	0.08	0.03	0.15	2.30	**0.008**	—	—	—	—	0.02	0.02	0.06	0.99	0.289	—	—	—	—
Clinical	−0.67	0.62	−0.09	−1.05	0.284	—	—	—	—	0.00	0.42	0.00	−0.01	0.994	—	—	—	—
PSWQ	−0.04	0.02	−0.12	−1.46	0.135	—	—	—	—	−0.04	0.02	−0.18	−2.22	**0.010**	—	—	—	—
Gender × Clinical	−0.40	1.18	−0.03	−0.30	0.715	—	—	—	—	−0.57	0.87	−0.07	−0.66	0.461	—	—	—	—
Gender × PSWQ	0.09	0.05	0.16	1.74	**0.048**	—	—	—	—	0.07	0.03	0.20	2.19	**0.003**	—	—	—	—

*Note*. *p* < 0.05 are printed in bold. Gender (0 = female, 1 = male); age in years; clinical (0 = nonclinical, 1 = clinical). *N* = 246.

Abbreviations: CRN, correct-response negativity; ERN, error-related negativity; PSWQ, Penn State Worry Questionnaire.

## Data Availability

The dataset and analytic code used to support the findings of this study have been deposited in the OSF repository (https://doi.org/10.17605/OSF.IO/7DCM3).

## Supporting Information

Supporting Information Figure S1: output with Specifications of the A Priori Sample Size Calculation Using G^*∗*^Power, version 3.1.9.6. Figure S2: grand-Averaged Waveforms and Corresponding Topographies for Error and Correct Trials of the Clinical Groups and the Healthy Control Group. Table S1: decomposition of Current Clinical Diagnoses across the Diagnostic Groups. Table S2: demographical, Questionnaire, and Clinical Data of the Clinical and Family Risk Groups. Table S3: electrophysiological and Behavioral Data of the Clinical and Family Risk Groups. Table S4: ANCOVA Results for ERN and CRN across Diagnostic Groups Using Different Scoring Approaches. Table S5: ANCOVA Results for ERN and CRN across Diagnostic Groups Including the Healthy Control Group Using Different Scoring Approaches. Table S6: ANCOVA Results of the Effects of Clinical Status of an Internalizing Disorder and Family Risk for Internalizing Psychopathology on the ERN and CRN Using Different Scoring Approaches. Table S7: the Role of Clinical Status, PSWQ, and MASQ-AA on the ERN and CRN within the Combined Sample across the Severity Continuum. Table S8: the Role of PSWQ and MASQ-AA on the ERN and CRN Using Different Scoring Approaches. Table S9: the Role of Gender and PSWQ on the ERN and CRN within the Combined Sample across the Severity Continuum.
