# Supplementary material for: Enhanced Performance Monitoring as a Transdiagnostic Risk Marker of the Anxiety and Obsessive–Compulsive Spectrum: The Role of Disorder Category, Clinical Status, Family Risk, and Anxiety Dimensions
Source: Depress Anxiety. 2025 Apr 13;2025:9505414. doi: 10.1155/da/9505414 (PMC12009682; doi:10.1155/da/9505414)
Supplement: Supporting Information — Figure S1: output with Specifications of the A Priori Sample Size Calculation Using G∗Power, version 3.1.9.6. Figure S2: grand-Averaged Waveforms and Corresponding Topographies for Error and Correct Trials of the Clinical Groups and the Healthy Control Group. Table S1: decomposition of Current Clinical Diagnoses across the Diagnostic Groups. Table S2: demographical, Questionnaire, and Clinical Data of the Clinical and Family Risk Groups. Table S3: electrophysiological and Behavioral Data of the Clinical and Family Risk Groups. Table S4: ANCOVA Results for ERN and CRN across Diagnostic Groups Using Different Scoring Approaches. Table S5: ANCOVA Results for ERN and CRN across Diagnostic Groups Including the Healthy Control Group Using Different Scoring Approaches. Table S6: ANCOVA Results of the Effects of Clinical Status of an Internalizing Disorder and Family Risk for Internalizing Psychopathology on the ERN and CRN Using Different Scoring Approaches. Table S7: the Role of Clinical Status, PSWQ, and MASQ-AA on the ERN and CRN within the Combined Sample across the Severity Continuum. Table S8: the Role of PSWQ and MASQ-AA on the ERN and CRN Using Different Scoring Approaches. Table S9: the Role of Gender and PSWQ on the ERN and CRN within the Combined Sample across the Severity Continuum. [file 9505414.f1.docx]

**SUPPLEMENTARY MATERIAL**

**Figures**

**Figure S1**

*Output with Specifications of the A Priori Sample Size Calculation Using G*Power, version 3.1.9.6.*

**
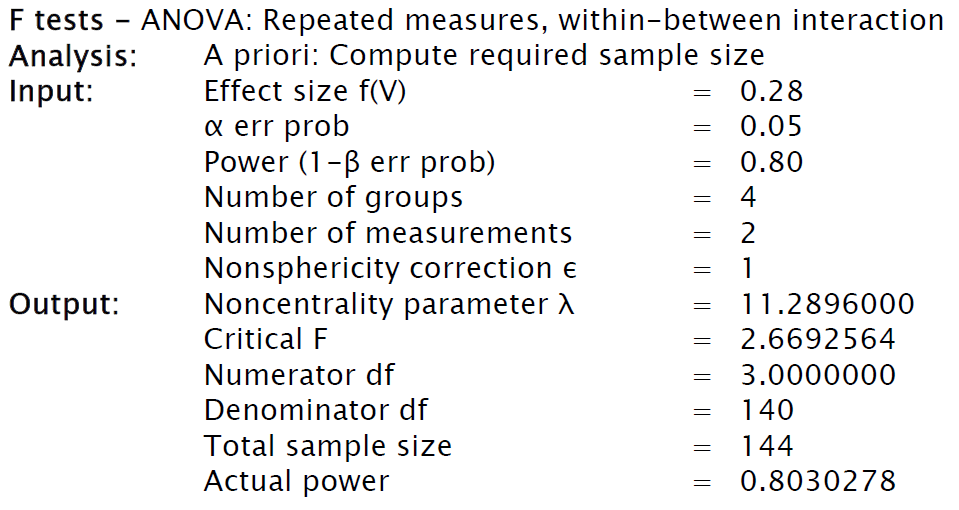
**

**Figure S2**

*Grand-Averaged Waveforms and Corresponding Topographies for Error and Correct Trials of the Clinical Groups and the Healthy Control Group.*


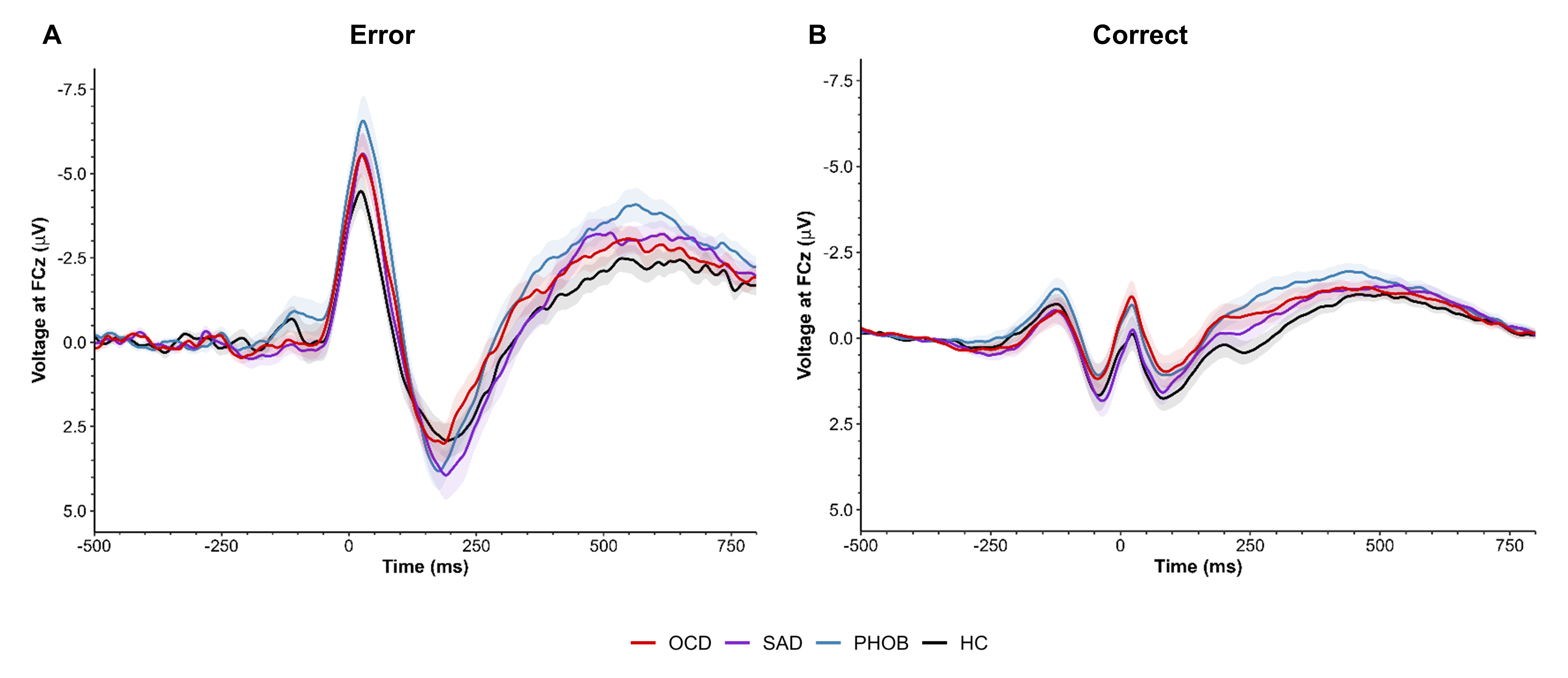


*Note.* Response-locked grand-averaged waveforms of erroneous and correct trials of each group (A, B). OCD = Obsessive-Compulsive Disorder; SAD = Social Anxiety Disorder; PHOB = Specific Phobia; HC = Healthy Control Group. *N* = 151.

**Tables**

**Table S1**

*Decomposition of Current Clinical Diagnoses across the Diagnostic Groups.*

|  | **Overall (*n*=156)** | OCD (*n*=39) | SAD (*n*=39) | PHOB (*n*=39) | CON (*n*=39) | Comparison | | |
| --- | --- | --- | --- | --- | --- | --- | --- | --- |
|  | ***N*** | *n* | *n* | *n* | *n* | *χ^2^* | *df* | *p* |
| Depression | **64** | 22 | 25 | 15 | 2 | 33.28 | 3 | **<.001** |
| Specific Phobia | **49** | 5 | 5 | 39 | 0 | 115.54 | 3 | **<.001** |
| Social Anxiety Disorder | **46** | 6 | 39 | 1 | 0 | 126.90 | 3 | **<.001** |
| Obsessive-Compulsive Disorder | **40** | 39 | 1 | 0 | 0 | 150.89 | 3 | **<.001** |
| Sleeping Disorder | **20** | 9 | 4 | 6 | 1 | 7.80 | 3 | .050 |
| Post-Traumatic Stress Disorder | **11** | 3 | 5 | 2 | 1 | 3.42 | 3 | .331 |
| Generalized Anxiety Disorder | **10** | 3 | 4 | 3 | 0 | 3.85 | 3 | .279 |
| Agoraphobia | **6** | 2 | 3 | 1 | 0 | 3.47 | 3 | .325 |
| Somatisation & Health Anxiety | **5** | 4 | 1 | 0 | 0 | 8.89 | 3 | **.031** |
| Panic Disorder | **5** | 2 | 1 | 2 | 0 | 2.27 | 3 | .518 |
| Tic Spectrum | **4** | 0 | 3 | 0 | 1 | 6.16 | 3 | .104 |
| Externalizing Spectrum | **4** | 0 | 2 | 2 | 0 | 4.11 | 3 | .250 |
| Eating Disorder & Body Dysmorphia | **3** | 3 | 0 | 0 | 0 | 9.18 | 3 | **.027** |
| Separation Anxiety | **1** | 0 | 1 | 0 | 0 | 3.02 | 3 | .389 |
| **Total Number of Diagnoses** | **268** | **98** | **94** | **71** | **5** |  |  |  |

*Note*. Sorted by overall frequency; obsessive-compulsive Disorder (OCD), social anxiety disorder (SAD), specific phobia (PHOB), control group (CON); tic spectrum includes skin picking, trichotillomania, and hoarding; externalizing spectrum includes attention deficit hyperactivity disorder and intermittent explosive disorder. *N* = 156.

*p* <.05 printed in bold

**Table S2**

*Demographical, Questionnaire, and Clinical Data of the Clinical and Family Risk Groups.*

|  | Nonclinical +  No Risk (*n* = 19) | |  | Nonclinical +  Risk (*n* = 15) | |  | Clinical +  No Risk (*n* = 34) | |  | Clinical +  Risk  (*n* =88) | |  | Group Comparison | | | |
| --- | --- | --- | --- | --- | --- | --- | --- | --- | --- | --- | --- | --- | --- | --- | --- | --- |
|  | *M* | *SD* |  | *M* | *SD* |  | *M* | *SD* |  | *M* | *SD* |  | *χ^2^ / F* | *df* | $\eta^{2}$ | *p* |
| Demographical |  |  |  |  |  |  |  |  |  |  |  |  |  |  |  |  |
| Gender (f/m) | 10/9 |  |  | 13/2 |  |  | 26/8 |  |  | 66/22 |  |  | 5.87 | 3 |  | .118 |
| Age | 30.32 | 8.53 |  | 30.60 | 8.36 |  | 29.35 | 10.25 |  | 27.44 | 6.85 |  | 1.30 | 3, 152 | 0.03 | .277 |
| Education | 12.32 | 1.00 |  | 12.00 | 1.00 |  | 11.97 | 1.31 |  | 12.31 | 0.88 |  | 1.19 | 3, 152 | 0.02 | .317 |
| Questionnaires |  |  |  |  |  |  |  |  |  |  |  |  |  |  |  |  |
| OCI-R | 4.16_a_ | 3.59 |  | 6.00_a_ | 5.68 |  | 10.68_a_ | 10.56 |  | 18.30_b_ | 13.25 |  | 12.36 | 3, 152 | 0.20 | **<.001** |
| LSAS-SR | 16.32_a_ | 9.98 |  | 17.53_a_ | 10.32 |  | 38.50_b_ | 28.55 |  | 53.21_b_ | 32.22 |  | 13.84 | 3, 151 | 0.22 | **<.001** |
| SMSP | 2.17_a_ | 3.13 |  | 0.60_a_ | 0.83 |  | 4.27_a_ | 4.91 |  | 8.80_b_ | 8.39 |  | 10.45 | 3, 150 | 0.17 | **<.001** |
| PSWQ | 34.63_a_ | 8.53 |  | 40.80_ab_ | 11.00 |  | 49.09_b_ | 13.57 |  | 57.10_c_ | 10.97 |  | 26.06 | 3, 152 | 0.34 | **<.001** |
| MASQ-AA | 19.42_a_ | 2.81 |  | 21.80_a_ | 4.33 |  | 24.00_a_ | 6.87 |  | 29.05_b_ | 9.37 |  | 10.76 | 3, 152 | 0.18 | **<.001** |
| STAI-T | 32.63_a_ | 6.99 |  | 35.33_ab_ | 7.68 |  | 42.79_b_ | 12.69 |  | 50.53_c_ | 12.07 |  | 18.42 | 3, 152 | 0.27 | **<.001** |
| STAI-S | 31.53_a_ | 6.46 |  | 32.86_a_ | 5.64 |  | 38.33_ab_ | 11.04 |  | 43.07_b_ | 12.45 |  | 8.05 | 3, 150 | 0.14 | **<.001** |
| BDI-II | 3.16_a_ | 4.37 |  | 2.80_a_ | 4.16 |  | 9.79_a_ | 11.07 |  | 15.55_b_ | 11.83 |  | 12.05 | 3, 152 | 0.19 | **<.001** |
| AUDIT | 3.74 | 2.79 |  | 4.00 | 3.64 |  | 4.29 | 3.14 |  | 4.06 | 3.19 |  | 0.13 | 3, 152 | 0.00 | .943 |
| EHI | 72.89 | 38.56 |  | 84.67 | 14.82 |  | 73.24 | 34.79 |  | 73.18 | 38.97 |  | 0.45 | 3, 152 | 0.01 | .717 |
| Clinical |  |  |  |  |  |  |  |  |  |  |  |  |  |  |  |  |
| Medication (y/n) | 0/19 |  |  | 0/15 |  |  | 2/32 |  |  | 13/75 |  |  | 6.86 | 3 |  | .077 |
| Diagnoses | 0.00_a_ | 0.00 |  | 0.00_a_ | 0.00 |  | 1.91_b_ | 1.58 |  | 2.82_c_ | 1.81 |  | 26.88 | 3, 152 | 0.35 | **<.001** |
| CGI-S | 0.95_a_ | 0.62 |  | 0.87_a_ | 0.52 |  | 2.82_b_ | 0.90 |  | 3.41_c_ | 1.08 |  | 55.67 | 3, 151 | 0.53 | **<.001** |
| GAF | 90.42_a_ | 4.44 |  | 87.67_a_ | 7.16 |  | 70.62_b_ | 13.77 |  | 61.76_c_ | 13.45 |  | 40.73 | 3, 151 | 0.45 | **<.001** |

*Note*. Gender (f = female, m = male); age and education in years; PSWQ = Penn State Worry Questionnaire; MASQ-AA = Mood and Anxiety Symptom Questionnaire (Anxious Arousal Subscale); OCI-R = Obsessive-Compulsive Inventory; LSAS-SR = Liebowitz Social Anxiety Scale – self report; SMSP = Severity Measure for Specific Phobia; STAI = State-Trait-Anxiety Inventory (Trait and State Subscale); BDI-II = Beck Depression Inventory II; AUDIT = Alcohol Use Disorders Identification Test; EHI = Edinburgh Handedness Inventory (Modified); Medication (y = yes, n = no) includes psychopharmacological treatment with antidepressants as reported by the participants; Diagnoses include index and comorbid diagnoses (*n*); CGI-S = Clinical Global Impression – Severity of Illness; GAF = Global Assessment of Functioning; degrees of freedom are deviating for some questionnaires due to missing data; means with different subscripts within rows indicate significant differences according to Sidak corrected post-hoc *t*-tests with *p* <.05. *N* = 156.

*p* < .05 are printed in bold.

**Table S3**

*Electrophysiological and Behavioral Data of the Clinical and Family Risk Groups.*

|  | Nonclinical + No Risk (*n* = 19) | |  | Nonclinical + Risk (*n* = 15) | |  | Clinical +  No Risk (*n* = 34) | |  | Clinical +  Risk  (*n* =88) | |
| --- | --- | --- | --- | --- | --- | --- | --- | --- | --- | --- | --- |
|  | *M* | *SD* |  | *M* | *SD* |  | *M* | *SD* |  | *M* | *SD* |
| ERPs |  |  |  |  |  |  |  |  |  |  |  |
| ERN (μV) | -1.94 | 2.78 |  | -3.40 | 2.38 |  | -3.26 | 3.06 |  | -4.34 | 3.88 |
| CRN (μV) | 1.29 | 1.90 |  | 0.43 | 2.65 |  | 0.75 | 2.08 |  | 0.00 | 2.60 |
| Behavior |  |  |  |  |  |  |  |  |  |  |  |
| Accuracy (%) | 86.26 | 7.39 |  | 83.65 | 12.77 |  | 87.18 | 7.18 |  | 84.94 | 8.28 |
| RT correct (ms) | 428.59 | 42.49 |  | 424.73 | 54.09 |  | 421.29 | 44.54 |  | 424.57 | 44.75 |
| RT incorrect (ms) | 386.85 | 69.93 |  | 366.40 | 61.29 |  | 357.91 | 64.06 |  | 355.01 | 53.18 |
| PES (ms) | 27.04 | 21.44 |  | 48.71 | 30.85 |  | 37.63 | 25.05 |  | 43.51 | 26.59 |

*Note*. ERPs = event-related potentials; ERN = error-related negativity (mean amplitude 0 – 100 ms at FCz); CRN = correct-response negativity (mean amplitude 0 – 100 ms at FCz); RT = response time; PES = post-error slowing. *N* = 156.

**Table S4**

*ANCOVA Results for ERN and CRN across Diagnostic Groups Using Different Scoring Approaches.*

|  | Response | | | |  | Group | | | |  | Response × Group | | | |
| --- | --- | --- | --- | --- | --- | --- | --- | --- | --- | --- | --- | --- | --- | --- |
| Variable | *F* | *df* | $\eta_{p}^{2}$ | *p* |  | *F* | *df* | $\eta_{p}^{2}$ | *p* |  | *F* | *df* | $\eta_{p}^{2}$ | *p* |
| Baseline -500 to -300ms |  |  |  |  |  |  |  |  |  |  |  |  |  |  |
| Mean Amplitude | 19.89 | 1, 151 | 0.12 | **<.001** |  | 1.56 | 3, 151 | 0.03 | .201 |  | 0.95 | 3, 151 | 0.02 | .418 |
| Adaptive Mean | 19.06 | 1, 151 | 0.11 | **<.001** |  | 1.51 | 3, 151 | 0.03 | .214 |  | 0.63 | 3, 151 | 0.01 | .598 |
| Baseline -200 to 0ms |  |  |  |  |  |  |  |  |  |  |  |  |  |  |
| Mean Amplitude | 24.18 | 1, 151 | 0.14 | **<.001** |  | 1.60 | 3, 151 | 0.03 | .192 |  | 0.71 | 3, 151 | 0.01 | .546 |
| Adaptive Mean | 23.90 | 1, 151 | 0.14 | **<.001** |  | 1.85 | 3, 151 | 0.04 | .141 |  | 0.53 | 3, 151 | 0.01 | .661 |
| Baseline Independent |  |  |  |  |  |  |  |  |  |  |  |  |  |  |
| Peak-to-Peak | 10.63 | 1, 151 | 0.07 | **.001** |  | 0.80 | 3, 151 | 0.02 | .497 |  | 0.63 | 3, 151 | 0.01 | .595 |

*Note*. ERN = error-related negativity; CRN = correct-response negativity; groups (OCD, SAD, PHOB, CON); response (correct, incorrect); mean amplitude (0 – 100 ms); adaptive mean around the peak (± 20 ms); peak-to-peak (difference between most positive pre-response and most negative post-response peak); all quantifications were based on electrode FCz; the ANCOVA included the covariate error response time, as this variable differed between the PHOB and CON group. *N* = 156.

*p* < .05 are printed in bold

**Table S5**

*ANCOVA Results for ERN and CRN across Diagnostic Groups Including the Healthy Control Group Using Different Scoring Approaches.*

|  | Response | | | |  | Group | | | |  | Response × Group | | | |
| --- | --- | --- | --- | --- | --- | --- | --- | --- | --- | --- | --- | --- | --- | --- |
| Variable | *F* | *df* | $\eta_{p}^{2}$ | *p* |  | *F* | *df* | $\eta_{p}^{2}$ | *p* |  | *F* | *df* | $\eta_{p}^{2}$ | *p* |
| Baseline -500 to -300ms |  |  |  |  |  |  |  |  |  |  |  |  |  |  |
| Mean Amplitude | 18.05 | 1, 146 | 0.11 | **<.001** |  | 2.49 | 3, 146 | 0.04 | .062 |  | 1.03 | 3, 146 | 0.02 | .379 |
| Adaptive Mean | 17.55 | 1, 146 | 0.11 | **<.001** |  | 2.55 | 3, 146 | 0.05 | .058 |  | 0.79 | 3, 146 | 0.02 | .504 |
| Baseline -200 to 0ms |  |  |  |  |  |  |  |  |  |  |  |  |  |  |
| Mean Amplitude | 22.29 | 1, 146 | 0.13 | **<.001** |  | 2.45 | 3, 146 | 0.05 | .066 |  | 0.86 | 3, 146 | 0.02 | .462 |
| Adaptive Mean | 22.39 | 1, 146 | 0.13 | **<.001** |  | 3.00 | 3, 146 | 0.06 | **.033** |  | 0.76 | 3, 146 | 0.02 | .520 |
| Baseline Independent |  |  |  |  |  |  |  |  |  |  |  |  |  |  |
| Peak-to-Peak | 10.00 | 1, 146 | 0.06 | **.002** |  | 1.34 | 3, 146 | 0.03 | .265 |  | 0.76 | 3, 146 | 0.02 | .517 |

*Note*. ERN = error-related negativity; CRN = correct-response negativity; groups (OCD, SAD, PHOB, HC); response (correct, incorrect); mean amplitude (0 – 100 ms); adaptive mean around the peak (± 20 ms); peak-to-peak (difference between most positive pre-response and most negative post-response peak); all quantifications were based on electrode FCz; the ANCOVA included the covariate error response time, as this variable differed between the PHOB and HC group. *N* = 151.

*p* < .05 are printed in bold

**Table S6**

*ANCOVA Results of the Effects of Clinical Status of an Internalizing Disorder and Family Risk for Internalizing Psychopathology on the ERN and CRN Using Different Scoring Approaches.*

|  | *F* | *df* | $\eta_{p}^{2}$ | *p* |
| --- | --- | --- | --- | --- |
| **Baseline -500 to -300ms** |  |  |  |  |
| **Mean Amplitude** |  |  |  |  |
| Response | 19.10 | 1, 150 | 0.11 | **<.001** |
| Response × Clinical | 0.69 | 1, 150 | 0.01 | .406 |
| Response × FHS Internalizing | 0.54 | 1, 150 | 0.00 | .466 |
| Response × Clinical × FHS Internalizing | 0.03 | 1, 150 | 0.00 | .860 |
| Clinical | 3.32 | 1, 150 | 0.02 | .071 |
| Clinical × FHS Internalizing | 0.12 | 1, 150 | 0.00 | .731 |
| FHS Internalizing | 4.35 | 1, 150 | 0.03 | **.039** |
| **Adaptive Mean** |  |  |  |  |
| Response | 18.37 | 1, 150 | 0.11 | **<.001** |
| Response × Clinical | 1.18 | 1, 150 | 0.01 | .278 |
| Response × FHS Internalizing | 0.08 | 1, 150 | 0.00 | .780 |
| Response × Clinical × FHS Internalizing | 0.02 | 1, 150 | 0.00 | .882 |
| Clinical | 4.01 | 1, 150 | 0.03 | **.047** |
| Clinical × FHS Internalizing | 0.05 | 1, 150 | 0.00 | .822 |
| FHS Internalizing | 2.78 | 1, 150 | 0.02 | .098 |
| **Baseline -200 to 0ms** |  |  |  |  |
| **Mean Amplitude** |  |  |  |  |
| Response | 23.26 | 1, 150 | 0.13 | **<.001** |
| Response × Clinical | 1.03 | 1, 150 | 0.01 | .313 |
| Response × FHS Internalizing | 0.13 | 1, 150 | 0.00 | .719 |
| Response × Clinical × FHS Internalizing | 0.26 | 1, 150 | 0.00 | .613 |
| Clinical | 6.40 | 1, 150 | 0.04 | **.012** |
| Clinical × FHS Internalizing | 0.02 | 1, 150 | 0.00 | .902 |
| FHS Internalizing | 0.03 | 1, 150 | 0.00 | .861 |
| **Adaptive Mean** |  |  |  |  |
| Response | 22.95 | 1, 150 | 0.13 | **<.001** |
| Response × Clinical | 1.64 | 1, 150 | 0.01 | .203 |
| Response × FHS Internalizing | 0.41 | 1, 150 | 0.00 | .524 |
| Response × Clinical × FHS Internalizing | 0.21 | 1, 150 | 0.00 | .651 |
| Clinical | 8.69 | 1, 150 | 0.06 | **.004** |
| Clinical × FHS Internalizing | 0.07 | 1, 150 | 0.00 | .791 |
| FHS Internalizing | 0.35 | 1, 150 | 0.00 | .553 |
| **Baseline Independent** |  |  |  |  |
| **Peak-to-Peak** |  |  |  |  |
| Response | 10.57 | 1, 150 | 0.07 | **.001** |
| Response × Clinical | 1.99 | 1, 150 | 0.01 | .160 |
| Response × FHS Internalizing | 1.08 | 1, 150 | 0.01 | .300 |
| Response × Clinical × FHS Internalizing | 0.92 | 1, 150 | 0.01 | .340 |
| Clinical | 4.85 | 1, 150 | 0.03 | **.029** |
| Clinical × FHS Internalizing | 0.20 | 1, 150 | 0.00 | .652 |
| FHS Internalizing | 2.99 | 1, 150 | 0.02 | .086 |

*Note*. ERN = error-related negativity; CRN = correct-response negativity; FHS = Family History Screen (no risk, risk); groups (nonclinical, clinical); response (correct, incorrect); mean amplitude (0 – 100 ms); adaptive mean around the peak (± 20 ms); peak-to-peak (difference between most positive pre-response and most negative post-response peak); all quantifications were based on electrode FCz. Analyses were controlled for error response time and post-error slowing, as these performance variables differed between the respective groups. *N* = 156.

*p* < .05 are printed in bold

**Table S7**

*The Role of Clinical Status, PSWQ, and MASQ-AA on the ERN and CRN within the Combined Sample across the Severity Continuum.*

|  | ERN | | | | | | | | |  | CRN | | | | | | | | |
| --- | --- | --- | --- | --- | --- | --- | --- | --- | --- | --- | --- | --- | --- | --- | --- | --- | --- | --- | --- |
|  | *b* | *SE_boot._* | *β* | *t* | *p_boot._* | *R^2^_corr_* | *F* | *df* | *p* |  | *b* | *SE_boot._* | *β* | *t* | *p_boot._* | *R^2^_corr_* | *F* | *df* | *p* |
| **Baseline -500 to -300ms** |  |  |  |  |  |  |  |  |  |  |  |  |  |  |  |  |  |  |  |
| **Mean Amplitude** |  |  |  |  |  | 0.02 | 1.62 | 9, 236 | .110 |  |  |  |  |  |  | 0.08 | 3.35 | 9, 236 | **<.001** |
| Gender | 0.78 | 0.61 | 0.09 | 1.33 | .198 |  |  |  |  |  | 1.08 | 0.34 | 0.18 | 2.85 | **.002** |  |  |  |  |
| Age | 0.07 | 0.03 | 0.13 | 1.97 | **.017** |  |  |  |  |  | 0.02 | 0.02 | 0.06 | 0.87 | .320 |  |  |  |  |
| Clinical | -1.07 | 0.59 | -0.14 | -1.70 | .069 |  |  |  |  |  | 0.05 | 0.41 | 0.01 | 0.13 | .907 |  |  |  |  |
| PSWQ | -0.04 | 0.05 | -0.13 | -0.89 | .366 |  |  |  |  |  | -0.06 | 0.03 | -0.28 | -1.90 | .060 |  |  |  |  |
| MASQ-AA | 0.03 | 0.06 | 0.07 | 0.48 | .569 |  |  |  |  |  | -0.01 | 0.04 | -0.04 | -0.31 | .766 |  |  |  |  |
| Clinical × PSWQ | 0.04 | 0.06 | 0.10 | 0.71 | .476 |  |  |  |  |  | 0.08 | 0.04 | 0.27 | 2.12 | **.032** |  |  |  |  |
| Clinical × MASQ-AA | -0.09 | 0.08 | -0.17 | -1.16 | .244 |  |  |  |  |  | -0.02 | 0.05 | -0.06 | -0.42 | .668 |  |  |  |  |
| PSWQ × MASQ-AA | -0.01 | 0.01 | -0.20 | -1.36 | .294 |  |  |  |  |  | 0.00 | 0.00 | -0.15 | -1.04 | .351 |  |  |  |  |
| Clinical × PSWQ × MASQ-AA | 0.01 | 0.01 | 0.24 | 1.54 | .194 |  |  |  |  |  | 0.00 | 0.00 | 0.01 | 0.05 | .969 |  |  |  |  |
| **Adaptive Mean** |  |  |  |  |  | 0.03 | 1.96 | 9, 236 | **.044** |  |  |  |  |  |  | 0.10 | 4.03 | 9, 236 | **<.001** |
| Gender | 0.84 | 0.61 | 0.09 | 1.33 | .174 |  |  |  |  |  | 1.34 | 0.40 | 0.21 | 3.26 | **.002** |  |  |  |  |
| Age | 0.10 | 0.03 | 0.16 | 2.45 | **.004** |  |  |  |  |  | 0.02 | 0.02 | 0.05 | 0.79 | .397 |  |  |  |  |
| Clinical | -1.30 | 0.64 | -0.15 | -1.93 | **.043** |  |  |  |  |  | 0.22 | 0.43 | 0.04 | 0.51 | .598 |  |  |  |  |
| PSWQ | -0.03 | 0.05 | -0.10 | -0.69 | .491 |  |  |  |  |  | -0.07 | 0.03 | -0.32 | -2.18 | **.037** |  |  |  |  |
| MASQ-AA | 0.05 | 0.06 | 0.09 | 0.68 | .421 |  |  |  |  |  | -0.02 | 0.05 | -0.06 | -0.42 | .692 |  |  |  |  |
| Clinical × PSWQ | 0.03 | 0.06 | 0.07 | 0.56 | .545 |  |  |  |  |  | 0.08 | 0.04 | 0.26 | 2.06 | **.040** |  |  |  |  |
| Clinical × MASQ-AA | -0.13 | 0.09 | -0.21 | -1.46 | .127 |  |  |  |  |  | -0.01 | 0.06 | -0.03 | -0.19 | .864 |  |  |  |  |
| PSWQ × MASQ-AA | -0.01 | 0.01 | -0.18 | -1.27 | .255 |  |  |  |  |  | -0.01 | 0.01 | -0.21 | -1.52 | .225 |  |  |  |  |
| Clinical × PSWQ × MASQ-AA | 0.01 | 0.01 | 0.27 | 1.72 | .120 |  |  |  |  |  | 0.00 | 0.01 | 0.05 | 0.33 | .776 |  |  |  |  |
| **Baseline -200 to 0ms** |  |  |  |  |  |  |  |  |  |  |  |  |  |  |  |  |  |  |  |
| **Mean Amplitude** |  |  |  |  |  | 0.03 | 1.69 | 9, 236 | .091 |  |  |  |  |  |  | 0.05 | 2.46 | 9, 236 | **.011** |
| Gender | 0.42 | 0.45 | 0.06 | 0.96 | .360 |  |  |  |  |  | 0.73 | 0.31 | 0.16 | 2.39 | **.018** |  |  |  |  |
| Age | 0.05 | 0.02 | 0.13 | 1.96 | **.017** |  |  |  |  |  | -0.01 | 0.02 | -0.03 | -0.46 | .610 |  |  |  |  |
| Clinical | -1.04 | 0.45 | -0.18 | -2.25 | **.020** |  |  |  |  |  | -0.02 | 0.31 | -0.01 | -0.06 | .949 |  |  |  |  |
| PSWQ | -0.02 | 0.03 | -0.10 | -0.69 | .481 |  |  |  |  |  | -0.05 | 0.02 | -0.30 | -2.02 | **.022** |  |  |  |  |
| MASQ-AA | 0.03 | 0.04 | 0.08 | 0.59 | .452 |  |  |  |  |  | 0.02 | 0.03 | 0.10 | 0.72 | .385 |  |  |  |  |
| Clinical × PSWQ | 0.02 | 0.04 | 0.06 | 0.48 | .619 |  |  |  |  |  | 0.07 | 0.03 | 0.33 | 2.54 | **.007** |  |  |  |  |
| Clinical × MASQ-AA | -0.08 | 0.06 | -0.18 | -1.29 | .179 |  |  |  |  |  | -0.04 | 0.04 | -0.13 | -0.93 | .242 |  |  |  |  |
| PSWQ × MASQ-AA | -0.01 | 0.00 | -0.18 | -1.20 | .275 |  |  |  |  |  | 0.00 | 0.00 | -0.06 | -0.39 | .671 |  |  |  |  |
| Clinical × PSWQ × MASQ-AA | 0.01 | 0.01 | 0.25 | 1.60 | .116 |  |  |  |  |  | 0.00 | 0.00 | -0.08 | -0.48 | .566 |  |  |  |  |
| **Adaptive Mean** |  |  |  |  |  | 0.05 | 2.27 | 9, 236 | **.018** |  |  |  |  |  |  | 0.09 | 3.75 | 9, 236 | **<.001** |
| Gender | 0.48 | 0.47 | 0.07 | 1.00 | .306 |  |  |  |  |  | 1.00 | 0.32 | 0.21 | 3.25 | **.004** |  |  |  |  |
| Age | 0.08 | 0.02 | 0.17 | 2.60 | **.004** |  |  |  |  |  | -0.01 | 0.02 | -0.03 | -0.48 | .598 |  |  |  |  |
| Clinical | -1.27 | 0.52 | -0.20 | -2.52 | **.014** |  |  |  |  |  | 0.15 | 0.31 | 0.04 | 0.46 | .607 |  |  |  |  |
| PSWQ | -0.02 | 0.04 | -0.07 | -0.44 | .653 |  |  |  |  |  | -0.06 | 0.02 | -0.38 | -2.60 | **.002** |  |  |  |  |
| MASQ-AA | 0.04 | 0.05 | 0.12 | 0.86 | .329 |  |  |  |  |  | 0.02 | 0.03 | 0.07 | 0.54 | .499 |  |  |  |  |
| Clinical × PSWQ | 0.01 | 0.05 | 0.04 | 0.30 | .753 |  |  |  |  |  | 0.08 | 0.03 | 0.34 | 2.68 | **.004** |  |  |  |  |
| Clinical × MASQ-AA | -0.11 | 0.06 | -0.24 | -1.68 | .067 |  |  |  |  |  | -0.03 | 0.04 | -0.09 | -0.66 | .434 |  |  |  |  |
| PSWQ × MASQ-AA | -0.01 | 0.01 | -0.16 | -1.10 | .300 |  |  |  |  |  | 0.00 | 0.00 | -0.16 | -1.15 | .166 |  |  |  |  |
| Clinical × PSWQ × MASQ-AA | 0.01 | 0.01 | 0.29 | 1.84 | .069 |  |  |  |  |  | 0.00 | 0.00 | -0.02 | -0.11 | .887 |  |  |  |  |
| **Baseline Independent** |  |  |  |  |  |  |  |  |  |  |  |  |  |  |  |  |  |  |  |
| **Peak-to-Peak** |  |  |  |  |  | 0.07 | 2.95 | 9, 236 | **.002** |  |  |  |  |  |  | 0.05 | 2.32 | 9, 236 | **.016** |
| Gender | 0.85 | 0.51 | 0.10 | 1.61 | .110 |  |  |  |  |  | 0.66 | 0.30 | 0.14 | 2.19 | **.036** |  |  |  |  |
| Age | 0.08 | 0.03 | 0.16 | 2.52 | **.006** |  |  |  |  |  | 0.00 | 0.02 | 0.00 | 0.01 | .993 |  |  |  |  |
| Clinical | -1.85 | 0.56 | -0.25 | -3.27 | **.003** |  |  |  |  |  | -0.19 | 0.30 | -0.05 | -0.60 | .532 |  |  |  |  |
| PSWQ | 0.01 | 0.04 | 0.05 | 0.30 | .761 |  |  |  |  |  | -0.07 | 0.03 | -0.42 | -2.81 | **.020** |  |  |  |  |
| MASQ-AA | 0.04 | 0.05 | 0.08 | 0.62 | .518 |  |  |  |  |  | 0.05 | 0.03 | 0.20 | 1.43 | .107 |  |  |  |  |
| Clinical × PSWQ | 0.00 | 0.05 | -0.01 | -0.04 | .976 |  |  |  |  |  | 0.07 | 0.03 | 0.35 | 2.66 | **.014** |  |  |  |  |
| Clinical × MASQ-AA | -0.14 | 0.07 | -0.26 | -1.85 | .053 |  |  |  |  |  | -0.05 | 0.04 | -0.18 | -1.27 | .127 |  |  |  |  |
| PSWQ × MASQ-AA | -0.01 | 0.01 | -0.17 | -1.18 | .259 |  |  |  |  |  | -0.01 | 0.00 | -0.40 | -2.73 | **.010** |  |  |  |  |
| Clinical × PSWQ × MASQ-AA | 0.01 | 0.01 | 0.34 | 2.20 | **.037** |  |  |  |  |  | 0.01 | 0.00 | 0.31 | 1.97 | **.044** |  |  |  |  |

*Note*. ERN = error-related negativity; CRN = correct-response negativity; Gender (0 = female, 1 = male); age in years; clinical (0 = nonclinical, 1 = clinical); PSWQ = Penn State Worry Questionnaire (mean centered); MASQ-AA = Mood and Anxiety Symptom Questionnaire (mean centered); mean amplitude (0 – 100 ms); adaptive mean around the peak (± 20 ms); peak-to-peak (difference between most positive pre-response and most negative post-response peak); all quantifications were based on electrode FCz. *N* = 246.

*p* < .05 are printed in bold

**Table S8**

*The Role of PSWQ and MASQ-AA on the ERN and CRN Using Different Scoring Approaches.*

|  | ERN | | | | | | | | |  | CRN | | | | | | | | |
| --- | --- | --- | --- | --- | --- | --- | --- | --- | --- | --- | --- | --- | --- | --- | --- | --- | --- | --- | --- |
|  | *b* | *SE_boot._* | *β* | *t* | *p_boot._* | *R^2^_corr_* | *F* | *df* | *p* |  | *b* | *SE_boot_* | *β* | *t* | *p_boot._* | *R^2^_corr_* | *F* | *df* | *p* |
| **Baseline -500 to -300ms** |  |  |  |  |  |  |  |  |  |  |  |  |  |  |  |  |  |  |  |
| **Mean Amplitude** |  |  |  |  |  | 0.02 | 1.62 | 9, 236 | .110 |  |  |  |  |  |  | 0.08 | 3.35 | 9, 236 | **<.001** |
| Gender | 0.71 | 0.63 | 0.09 | 1.08 | .262 |  |  |  |  |  | 1.18 | 0.41 | 0.21 | 2.65 | **.007** |  |  |  |  |
| Age | 0.06 | 0.03 | 0.13 | 1.55 | **.042** |  |  |  |  |  | 0.01 | 0.02 | 0.02 | 0.24 | .793 |  |  |  |  |
| PSWQ | -0.04 | 0.03 | -0.16 | -1.47 | .090 |  |  |  |  |  | -0.01 | 0.02 | -0.03 | -0.29 | .749 |  |  |  |  |
| MASQ-AA | 0.03 | 0.04 | 0.06 | 0.51 | .494 |  |  |  |  |  | -0.03 | 0.03 | -0.09 | -0.74 | .378 |  |  |  |  |
| PSWQ × MASQ-AA | 0.00 | 0.00 | -0.03 | -0.33 | .637 |  |  |  |  |  | 0.00 | 0.00 | -0.10 | -1.01 | .167 |  |  |  |  |
| **Adaptive Mean** |  |  |  |  |  | 0.03 | 1.96 | 9, 236 | **.044** |  |  |  |  |  |  | 0.10 | 4.03 | 9, 236 | **<.001** |
| Gender | 0.81 | 0.64 | 0.09 | 1.13 | .222 |  |  |  |  |  | 1.42 | 0.49 | 0.23 | 2.91 | **.007** |  |  |  |  |
| Age | 0.08 | 0.03 | 0.16 | 1.95 | **.017** |  |  |  |  |  | 0.00 | 0.03 | -0.01 | -0.10 | .916 |  |  |  |  |
| PSWQ | -0.05 | 0.03 | -0.18 | -1.60 | .065 |  |  |  |  |  | -0.01 | 0.02 | -0.05 | -0.49 | .576 |  |  |  |  |
| MASQ-AA | 0.04 | 0.04 | 0.10 | 0.82 | .261 |  |  |  |  |  | -0.03 | 0.03 | -0.10 | -0.83 | .308 |  |  |  |  |
| PSWQ × MASQ-AA | 0.00 | 0.00 | -0.03 | -0.31 | .699 |  |  |  |  |  | 0.00 | 0.00 | -0.09 | -0.97 | .270 |  |  |  |  |
| **Baseline -200 to 0ms** |  |  |  |  |  |  |  |  |  |  |  |  |  |  |  |  |  |  |  |
| **Mean Amplitude** |  |  |  |  |  | 0.03 | 1.69 | 9, 236 | .091 |  |  |  |  |  |  | 0.05 | 2.46 | 9, 236 | **.011** |
| Gender | 0.14 | 0.46 | 0.02 | 0.30 | .752 |  |  |  |  |  | 0.73 | 0.41 | 0.15 | 1.78 | .068 |  |  |  |  |
| Age | 0.04 | 0.02 | 0.12 | 1.40 | .071 |  |  |  |  |  | -0.02 | 0.02 | -0.07 | -0.79 | .362 |  |  |  |  |
| PSWQ | -0.04 | 0.02 | -0.19 | -1.75 | .073 |  |  |  |  |  | 0.01 | 0.02 | 0.05 | 0.41 | .644 |  |  |  |  |
| MASQ-AA | 0.01 | 0.03 | 0.04 | 0.33 | .702 |  |  |  |  |  | -0.03 | 0.03 | -0.12 | -1.01 | .240 |  |  |  |  |
| PSWQ × MASQ-AA | 0.00 | 0.00 | 0.00 | 0.00 | .997 |  |  |  |  |  | 0.00 | 0.00 | -0.03 | -0.30 | .719 |  |  |  |  |
| **Adaptive Mean** |  |  |  |  |  | 0.05 | 2.27 | 9, 236 | **.018** |  |  |  |  |  |  | 0.09 | 3.75 | 9, 236 | **<.001** |
| Gender | 0.24 | 0.49 | 0.04 | 0.45 | .637 |  |  |  |  |  | 0.97 | 0.44 | 0.20 | 2.41 | **.036** |  |  |  |  |
| Age | 0.06 | 0.02 | 0.16 | 1.97 | **.015** |  |  |  |  |  | -0.03 | 0.02 | -0.10 | -1.19 | .197 |  |  |  |  |
| PSWQ | -0.04 | 0.02 | -0.21 | -1.92 | **.044** |  |  |  |  |  | 0.00 | 0.02 | 0.02 | 0.15 | .883 |  |  |  |  |
| MASQ-AA | 0.03 | 0.04 | 0.09 | 0.78 | .388 |  |  |  |  |  | -0.04 | 0.03 | -0.15 | -1.21 | .166 |  |  |  |  |
| PSWQ × MASQ-AA | 0.00 | 0.00 | 0.00 | 0.00 | .999 |  |  |  |  |  | 0.00 | 0.00 | -0.04 | -0.37 | .662 |  |  |  |  |
| **Baseline Independent** |  |  |  |  |  |  |  |  |  |  |  |  |  |  |  |  |  |  |  |
| **Peak-to-Peak** |  |  |  |  |  | 0.07 | 2.95 | 9, 236 | **.002** |  |  |  |  |  |  | 0.05 | 2.32 | 9, 236 | **.016** |
| Gender | 0.57 | 0.53 | 0.08 | 0.92 | .287 |  |  |  |  |  | 0.62 | 0.36 | 0.14 | 1.70 | .078 |  |  |  |  |
| Age | 0.07 | 0.03 | 0.16 | 1.94 | **.015** |  |  |  |  |  | -0.01 | 0.02 | -0.05 | -0.61 | .486 |  |  |  |  |
| PSWQ | -0.03 | 0.02 | -0.11 | -1.02 | .248 |  |  |  |  |  | 0.00 | 0.01 | -0.01 | -0.10 | .908 |  |  |  |  |
| MASQ-AA | 0.02 | 0.04 | 0.05 | 0.41 | .674 |  |  |  |  |  | 0.00 | 0.03 | -0.01 | -0.07 | .946 |  |  |  |  |
| PSWQ × MASQ-AA | 0.00 | 0.00 | 0.03 | 0.34 | .640 |  |  |  |  |  | 0.00 | 0.00 | -0.03 | -0.33 | .729 |  |  |  |  |

*Note*. ERN = error-related negativity; CRN = correct-response negativity; Gender (0 = female, 1 = male); age in years; PSWQ = Penn State Worry Questionnaire (mean centered); MASQ-AA = Mood and Anxiety Symptom Questionnaire (mean centered); mean amplitude (0 – 100 ms); adaptive mean around the peak (± 20 ms); peak-to-peak (difference between most positive pre-response and most negative post-response peak); all quantifications were based on electrode FCz. *N* = 156.

*p* < .05 are printed in bold

**Table S9**

*The Role of Gender and PSWQ on the ERN and CRN within the Combined Sample across the Severity Continuum.*

|  | ERN | | | | | | | | |  | CRN | | | | | | | | |
| --- | --- | --- | --- | --- | --- | --- | --- | --- | --- | --- | --- | --- | --- | --- | --- | --- | --- | --- | --- |
|  | *b* | *SE_boot._* | *β* | *t* | *p_boot._* | *R^2^_corr_* | *F* | *df* | *p* |  | *b* | *SE_boot_* | *β* | *t* | *p_boot._* | *R^2^_corr_* | *F* | *df* | *p* |
| **Baseline -500 to -300ms** |  |  |  |  |  |  |  |  |  |  |  |  |  |  |  |  |  |  |  |
| **Mean Amplitude** |  |  |  |  |  | 0.03 | 2.41 | 6, 239 | **.028** |  |  |  |  |  |  | 0.06 | 3.79 | 6, 239 | **.001** |
| Gender | 1.30 | 0.93 | 0.15 | 1.34 | .155 |  |  |  |  |  | 1.76 | 0.63 | 0.30 | 2.78 | **.002** |  |  |  |  |
| Age | 0.08 | 0.03 | 0.15 | 2.30 | **.008** |  |  |  |  |  | 0.02 | 0.02 | 0.06 | 0.99 | .289 |  |  |  |  |
| Clinical | -0.67 | 0.62 | -0.09 | -1.05 | .284 |  |  |  |  |  | 0.00 | 0.42 | 0.00 | -0.01 | .994 |  |  |  |  |
| PSWQ | -0.04 | 0.02 | -0.12 | -1.46 | .135 |  |  |  |  |  | -0.04 | 0.02 | -0.18 | -2.22 | **.010** |  |  |  |  |
| Gender × Clinical | -0.40 | 1.18 | -0.03 | -0.30 | .715 |  |  |  |  |  | -0.57 | 0.87 | -0.07 | -0.66 | .461 |  |  |  |  |
| Gender × PSWQ | 0.09 | 0.05 | 0.16 | 1.74 | **.048** |  |  |  |  |  | 0.07 | 0.03 | 0.20 | 2.19 | **.003** |  |  |  |  |
| **Adaptive Mean** |  |  |  |  |  | 0.05 | 2.98 | 6, 239 | **.008** |  |  |  |  |  |  | 0.08 | 4.40 | 6, 239 | **<.001** |
| Gender | 1.26 | 1.07 | 0.13 | 1.21 | .223 |  |  |  |  |  | 2.11 | 0.68 | 0.33 | 3.05 | **<.001** |  |  |  |  |
| Age | 0.11 | 0.03 | 0.19 | 2.88 | **<.001** |  |  |  |  |  | 0.02 | 0.03 | 0.05 | 0.81 | .397 |  |  |  |  |
| Clinical | -0.87 | 0.63 | -0.10 | -1.26 | .170 |  |  |  |  |  | 0.24 | 0.47 | 0.04 | 0.53 | .626 |  |  |  |  |
| PSWQ | -0.04 | 0.02 | -0.12 | -1.38 | .122 |  |  |  |  |  | -0.05 | 0.02 | -0.21 | -2.56 | **.016** |  |  |  |  |
| Gender × Clinical | -0.15 | 1.32 | -0.01 | -0.11 | .894 |  |  |  |  |  | -0.75 | 0.91 | -0.09 | -0.78 | .397 |  |  |  |  |
| Gender × PSWQ | 0.11 | 0.05 | 0.18 | 1.92 | **.039** |  |  |  |  |  | 0.07 | 0.03 | 0.18 | 2.00 | **.019** |  |  |  |  |
| **Baseline -200 to 0ms** |  |  |  |  |  |  |  |  |  |  |  |  |  |  |  |  |  |  |  |
| **Mean Amplitude** |  |  |  |  |  | 0.05 | 3.05 | 6, 239 | **.007** |  |  |  |  |  |  | 0.04 | 2.78 | 6, 239 | **.013** |
| Gender | 1.14 | 0.73 | 0.17 | 1.60 | .116 |  |  |  |  |  | 1.56 | 0.43 | 0.33 | 3.04 | **<.001** |  |  |  |  |
| Age | 0.07 | 0.02 | 0.16 | 2.51 | **.003** |  |  |  |  |  | -0.01 | 0.02 | -0.02 | -0.34 | .709 |  |  |  |  |
| Clinical | -0.62 | 0.44 | -0.11 | -1.31 | .157 |  |  |  |  |  | -0.04 | 0.31 | -0.01 | -0.12 | .910 |  |  |  |  |
| PSWQ | -0.03 | 0.02 | -0.15 | -1.75 | .058 |  |  |  |  |  | -0.02 | 0.01 | -0.12 | -1.44 | .129 |  |  |  |  |
| Gender × Clinical | -0.83 | 0.91 | -0.10 | -0.85 | .347 |  |  |  |  |  | -0.96 | 0.63 | -0.15 | -1.35 | .110 |  |  |  |  |
| Gender × PSWQ | 0.10 | 0.03 | 0.24 | 2.53 | **.003** |  |  |  |  |  | 0.06 | 0.02 | 0.22 | 2.30 | **.007** |  |  |  |  |
| **Adaptive Mean** |  |  |  |  |  | 0.07 | 3.83 | 6, 239 | **.001** |  |  |  |  |  |  | 0.07 | 4.15 | 6, 239 | **<.001** |
| Gender | 1.10 | 0.87 | 0.15 | 1.41 | .192 |  |  |  |  |  | 1.91 | 0.45 | 0.40 | 3.70 | **<.001** |  |  |  |  |
| Age | 0.09 | 0.02 | 0.21 | 3.27 | **<.001** |  |  |  |  |  | -0.01 | 0.02 | -0.03 | -0.46 | .588 |  |  |  |  |
| Clinical | -0.82 | 0.45 | -0.13 | -1.58 | .065 |  |  |  |  |  | 0.21 | 0.32 | 0.05 | 0.60 | .536 |  |  |  |  |
| PSWQ | -0.03 | 0.02 | -0.13 | -1.62 | .077 |  |  |  |  |  | -0.03 | 0.01 | -0.18 | -2.15 | **.046** |  |  |  |  |
| Gender × Clinical | -0.58 | 1.04 | -0.06 | -0.54 | .545 |  |  |  |  |  | -1.13 | 0.66 | -0.18 | -1.59 | .087 |  |  |  |  |
| Gender × PSWQ | 0.11 | 0.04 | 0.25 | 2.71 | **.006** |  |  |  |  |  | 0.06 | 0.03 | 0.21 | 2.28 | **.015** |  |  |  |  |
| **Baseline Independent** |  |  |  |  |  |  |  |  |  |  |  |  |  |  |  |  |  |  |  |
| **Peak-to-Peak** |  |  |  |  |  | 0.07 | 4.13 | 6, 239 | **<.001** |  |  |  |  |  |  | 0.03 | 2.24 | 6, 239 | **.040** |
| Gender | 1.21 | 0.87 | 0.15 | 1.38 | .167 |  |  |  |  |  | 1.37 | 0.42 | 0.30 | 2.72 | **.005** |  |  |  |  |
| Age | 0.10 | 0.03 | 0.20 | 3.20 | **<.001** |  |  |  |  |  | 0.00 | 0.02 | 0.00 | 0.07 | .932 |  |  |  |  |
| Clinical | -1.32 | 0.51 | -0.18 | -2.27 | **.015** |  |  |  |  |  | 0.09 | 0.36 | 0.02 | 0.26 | .818 |  |  |  |  |
| PSWQ | -0.02 | 0.02 | -0.07 | -0.85 | .328 |  |  |  |  |  | -0.02 | 0.01 | -0.14 | -1.64 | .136 |  |  |  |  |
| Gender × Clinical | -0.10 | 1.06 | -0.01 | -0.08 | .922 |  |  |  |  |  | -0.86 | 0.62 | -0.14 | -1.25 | .160 |  |  |  |  |
| Gender × PSWQ | 0.11 | 0.04 | 0.22 | 2.42 | **.006** |  |  |  |  |  | 0.06 | 0.02 | 0.22 | 2.32 | **.007** |  |  |  |  |

*Note.* ERN = error-related negativity; CRN = correct-response negativity; Gender (0 = female, 1 = male); age in years; clinical (0 = nonclinical, 1 = clinical); PSWQ = Penn State Worry Questionnaire (mean centered); adaptive mean around the peak (± 20 ms); peak-to-peak (difference between most positive pre-response and most negative post-response peak); all quantifications were based on electrode FCz. *N* = 246.

*p* < .05 are printed in bold
